# ATP1A3 regulates protein synthesis for mitochondrial stability under heat stress

**DOI:** 10.1242/dmm.050574

**Published:** 2024-07-02

**Authors:** Fumihiko Fujii, Hikaru Kanemasa, Sayaka Okuzono, Daiki Setoyama, Ryoji Taira, Kousuke Yonemoto, Yoshitomo Motomura, Hiroki Kato, Keiji Masuda, Takahiro A. Kato, Shouichi Ohga, Yasunari Sakai

**Affiliations:** ^1^ Department of Pediatrics, Graduate School of Medical Sciences, Kyushu University, Fukuoka, 812-8582, Japan; ^2^ Department of Clinical Chemistry and Laboratory Medicine, Graduate School of Medical Sciences, Kyushu University, Fukuoka, 812-8582, Japan; ^3^ Department of Molecular Cell Biology and Oral Anatomy, Graduate School of Dental Science, Kyushu University, Fukuoka, 812-8582, Japan; ^4^ Section of Oral Medicine for Children, Division of Oral Health, Growth and Development, Faculty of Dental Science, Kyushu University, Fukuoka, 812-8582, Japan; ^5^ Department of Neuropsychiatry, Graduate School of Medical Sciences, Kyushu University, Fukuoka, 812-8582, Japan

**Keywords:** Na^+^/K^+^-ATPase α3 subunit (ATP1A3), Alternating hemiplegia of childhood, Interaction, Heat stress, Protein synthesis, Mitochondria, Induced pluripotent stem cells (iPSCs)

## Abstract

Pathogenic variants in *ATP1A3*, the gene encoding the α3 subunit of the Na^+^/K^+^-ATPase, cause alternating hemiplegia of childhood (AHC) and related disorders. Impairments in Na^+^/K^+^-ATPase activity are associated with the clinical phenotype. However, it remains unclear whether additional mechanisms are involved in the exaggerated symptoms under stressed conditions in patients with AHC. We herein report that the intracellular loop (ICL) of ATP1A3 interacted with RNA-binding proteins, such as Eif4g (encoded by *Eif4g1*), Pabpc1 and Fmrp (encoded by *Fmr1*), in mouse Neuro2a cells. Both the siRNA-mediated depletion of *Atp1a3* and ectopic expression of the p.R756C variant of human ATP1A3-ICL in Neuro2a cells resulted in excessive phosphorylation of ribosomal protein S6 (encoded by *Rps6*) and increased susceptibility to heat stress. In agreement with these findings, induced pluripotent stem cells (iPSCs) from a patient with the p.R756C variant were more vulnerable to heat stress than control iPSCs. Neurons established from the patient-derived iPSCs showed lower calcium influxes in responses to stimulation with ATP than those in control iPSCs. These data indicate that inefficient protein synthesis contributes to the progressive and deteriorating phenotypes in patients with the p.R756C variant among a variety of *ATP1A3-*related disorders.

## Teaser

**Summary:** Pathogenic variants in *ATP1A3* cause alternating hemiplegia of childhood and related disorders. ATP1A3 supports the expression of heat shock proteins, thereby protecting cells from mitochondrial instability under heat stress.

**Research Simplified**

Alternating hemiplegia of childhood (AHC) is a rare neurodevelopmental disorder characterised by repeated attacks of paralysis that affect any one side of the body, abnormal eye movements, delayed psychomotor development and seizures. Mutations in a gene that codes for ATP1A3 - a highly expressed protein in the human brain - have been implicated in AHC and other neurological disorders. Understanding how ATP1A3 mutations cause AHC-related symptoms can help researchers develop more potent therapeutics for AHC.

First, the authors showed that human ATP1A3 interacts with several known RNA-binding proteins that are essential in gene regulation. Among these RNA-binding proteins, heat shock proteins that are essential for correct folding of proteins were poorly expressed in ATP1A3-deficient mouse neuronal cells. These cells displayed impaired protein production and were sensitive to heat stress. Next, they showed that human cells with AHC-associated mutations in the gene for ATP1A3 led to partial expression of ATP1A3 and a further reduction in heat shock proteins. Additionally, ATP1A3-deficient human neurons comprised shorter protrusions extending from the cells (dendrites) and could not generate a complete functional response to chemical triggers, similar to neuronal dysfunction seen in AHC patients.

This work showed that AHC-associated mutations in the gene for ATP1A3 lead to ATP1A3 deficiency in both humans and mice that further disrupts gene regulation and robust protein production. Further research into ATP1A3 variants can facilitate development of therapeutic targets for AHC and other ATP1A3-related neurological disorders.

## INTRODUCTION

Alternating hemiplegia of childhood (AHC) is a rare neurodevelopmental disorder characterized by recurrent attacks of hemiplegia, involuntary movements and moderate to severe delay in psychomotor development (Heinzen et al., 2014; Rosewich et al., 2012). Affected children also develop dysautonomic symptoms that may require emergency cardiopulmonary resuscitation. Although flunarizine is effective in decreasing the frequency and magnitude of hemiplegic attacks (Cordani et al., 2021), the medication does not necessarily prevent sudden unexpected death in epilepsy or cardiac arrhythmia (Jaffer et al., 2015). Thus, the long-term outcomes of AHC need to be improved.

AHC is associated with *de novo* mutations in *ATP1A3* (OMIM #614820) (Heinzen et al., 2014, 2012; Rosewich et al., 2012). The human *ATP1A3* gene, located on chromosome 19q13.2, encodes the α3 subunit of sodium/potassium (Na^+^/K^+^)-ATPase (Clausen et al., 2017). Increasing evidence shows that pathogenic variations in *ATP1A3* cause not only AHC, but also a broad spectrum of neurological dysfunctions, including rapid-onset dystonia parkinsonism; cerebellar ataxia, areflexia, *pes cavus* (feet with high arches), optic atrophy and sensorineural hearing loss (collectively known as CAPOS syndrome); and relapsing encephalopathy with cerebellar ataxia (RECA) (Heinzen et al., 2014, 2012; Rosewich et al., 2012). More recently, mutations in *ATP1A3* have been identified in patients with polymicrogyria (Miyatake et al., 2021), early forms of intellectual deficits with epilepsy and ataxia (Paciorkowski et al., 2015), childhood-onset schizophrenia (Smedemark-Margulies et al., 2016), and familial childhood-onset progressive cerebellar syndrome (Jaffer et al., 2017). The clinical features of these patients are correlated with the position of mutations in *ATP1A3* (Heinzen et al., 2014; Rosewich et al., 2012; Sasaki et al., 2014) and the biochemical activity of Na^+^/K^+^-ATPase (Blanco-Arias et al., 2009; Calame et al., 2023; Li et al., 2015; Moreno et al., 2022; Toustrup-Jensen et al., 2014; Tranebjaerg et al., 2018).

ATP1A3 is highly expressed in the human brain (McGrail et al., 1991). This protein is responsible for maintaining the electrochemical gradient across the cell membrane through the active transport of sodium and potassium ions (Holm et al., 2016a). Notably, molecular studies suggest that the Na^+^/K^+^-ATPase anchors functional molecules, thereby regulating the activity of downstream signaling pathways, such as the Ras-MAPK (Haas et al., 2002) and phosphatidylinositol trisphosphate receptor (Zhang et al., 2006) pathways. Thus, ATP1A3 might have versatile functions in the developing brain, which may clarify the distinct pathogenesis of AHC from that of other *ATP1A3*-related diseases.

Several reports have shown that the p.R756C variant in *ATP1A3* is associated with RECA (Biela et al., 2021; Kanemasa et al., 2016). Among *ATP1A3*-related disorders, RECA is characterized by recurrent generalized weakness, disturbed consciousness in febrile conditions and involuntary movements after recovery from acute illness (Dard et al., 2015; Kanemasa et al., 2016; Sabouraud et al., 2019). Deterioration in cognitive function and motor impairment are typically evident after the onset of neurological symptoms. However, the mechanisms underlying such thermolabile symptoms remain unclear.

In this study, we searched for functional molecules that could bind to the cytosolic domain of ATP1A3. We found that ATP1A3 physically interacts with various RNA-binding proteins and molecular chaperones. Our study is the first to show that the exacerbation of RECA, a rare subtype of *ATP1A3*-related diseases (Blanco-Arias et al., 2009; Calame et al., 2023; Li et al., 2015; Moreno et al., 2022; Toustrup-Jensen et al., 2014; Tranebjaerg et al., 2018), is associated with inefficient protein synthesis in cells expressing mutant ATP1A3.

## RESULTS

### ATP1A3 interacts with RNA-binding proteins

**Fig. 1.**
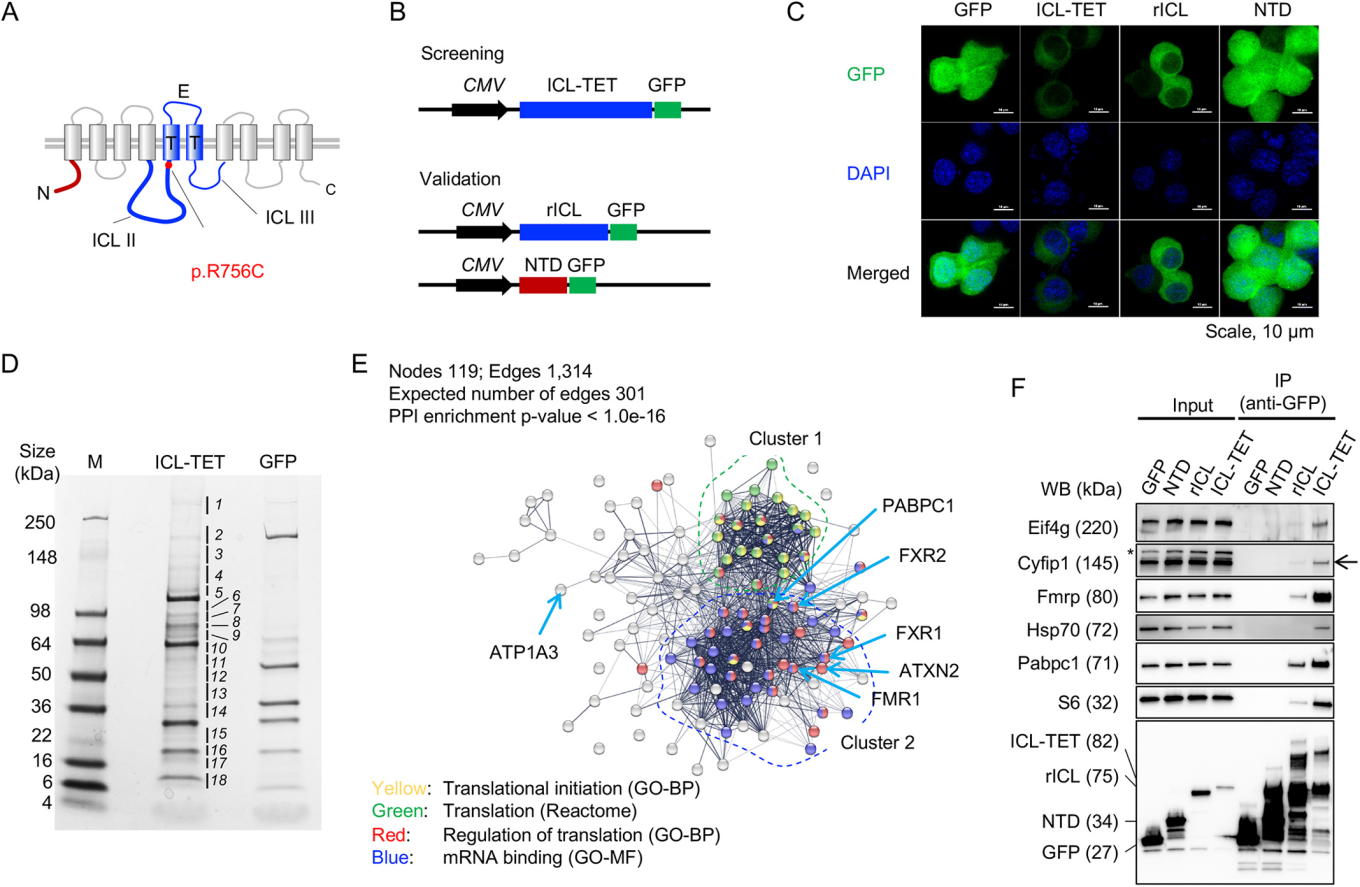
**The intracellular loop of ATP1A3 interacts with regulators of cap-dependent translation.** (A) Location of the functional domains and the R756C mutation in ATP1A3. The N-terminal domain (NTD, red), the second and the third intracellular loops (ICL II and III), transmembrane domains (‘T’, columns) and extracellular loop (‘E’) are shown in the schematic. Blue lines indicate the ICL-T-E-T (TET) region expressed in Neuro2a cells (used for proteomic screening). The thick blue line represents the refined ICL II domain (rICL; used for validation study). (B) Design of the plasmid expressing the ICL-TET-GFP (top), rICL-GFP (middle) and NTD-GFP (bottom) fusion proteins. ICL-TET-GFP was used for the initial proteomic study (screening) and the plasmids expressing rICL-GFP and NTD-GFP were used for the validation study. (C) Confocal microscopy image of Neuro2a cells expressing GFP, ICL-TET-GFP, rICL-GFP and NTD-GFP. (D) Coomassie Brilliant Blue staining of proteins co-immunoprecipitated with GFP and ICL-TET-GFP in Neuro2a cells. The indicated protein bands (1-18) were subjected to mass spectrometry. M, marker. (E) Interaction network for ATP1A3 (ICL-TET-GFP)-binding proteins. Gray and colored nodes represent the ICL-TET-GFP-binding proteins identified in this study. Clusters 1 and 2 (green and blue dashed lines) were enriched in distinctive categories of protein functions according to the Gene Ontology (GO), Reactome and KEGG databases. PPI, protein-protein interaction. (F) Western blotting (WB) for proteins co-immunoprecipitated with GFP, NTD-GFP rICL-GFP or ICL-TET-GFP. Asterisks, non-specific bands; arrow, CYFIP1 signal (145 kDa). Immunofluorescence images and western blots are representative of three independent experiments.

To identify ATP1A3-binding proteins, we used an EGFP-tagged human ATP1A3 fragment consisting of an intracellular loop (ICL), two transmembrane (T) domains and an extracellular loop (E) (amino acids T335 to L839 (see Materials and Methods), ICL-TET-GFP) as a bait fragment (Fig. 1A-C) and EGFP as a negative control. Proteins copurified with ICL-TET-GFP from mouse Neuro2a cells formed 18 unique bands as seen by Coomassie Brilliant Blue staining following SDS-PAGE (Fig. 1D). Trypsin-digested peptides were then subjected to mass spectrometry, which identified 1817 proteins as potential binding partners of ICL-TET. Among them, 136 proteins passed the filtering criterion of the reliability index being ≥50 (Table S1).

Using the Search Tool for the Retrieval of Interacting Genes/Proteins (STRING) database, we developed a protein–protein interaction network (Fig. 1E; Fig. S1). Gene Ontology (GO) and KEGG pathway analyses revealed that the following annotations were enriched in the network: ‘cytoplasmic translation’ [GO biological process (GO-BP) 0002181: *P*=3.71×10^−18^], ‘ribonucleoprotein complex’ [GO cellular component (GO-CC) 1990904: *P*=1.50×10^−51^], ‘RNA binding’ [GO molecular function (GO-MF) 0003723: *P*=1.58×10^−69^] and ‘ribosome’ (KEGG hsa03010: *P*=4.99×10^−13^) (Fig. S2). A Reactome analysis further detected the enrichment of molecular pathways related to ‘metabolism of RNA’ (R-HSA-8953854: *P*=9.20×10^−24^), ‘eukaryotic translation initiation’ (R-HSA-72613: *P*=1.63×10^−17^), and ‘cap-dependent translation initiation’ (R-HSA-72737: *P*=3.74×10^−17^) (Fig. S2). Therefore, we focused on the potential role of ATP1A3 in RNA translation.

Among the RNA-binding proteins, we found that fragile X messenger ribonucleoprotein (FMRP, encoded by *Fmr1*) and its functionally associated molecules were present in the network (Pasciuto and Bagni, 2014). Interestingly, the ataxia-related protein ATXN2 is also present among the ICL-interacting proteins (Naruse et al., 2019). Co-immunoprecipitation (Co-IP) and western blotting analyses validated the interactions of ATP1A3 (ICL) with Eif4g (encoded by *Eif4g1*), Cyfip1, Fmrp, Pabpc1 and ribosomal protein S6 (encoded by *Rps6*) (Fig. 1F; Fig. S3).

We found that the GFP-tagged refined ICL II domain (amino acids T329-L762; rICL-GFP) was more efficiently expressed than ICL-TET-GFP, the original ICL used as a bait for the proteomic screening (Fig. 1C,F). Thus, the C-terminally attached TET portion was considered to disturb the folding and expression of rICL in the cytosolic fraction. In agreement with this concept, Hsp70 was co-purified with ICL-TET-GFP but not with rICL-GFP. Thus, heat shock proteins (HSPs) are likely to bind to ICL-TET domains as cell-protective molecules against the toxic effects of misfolded proteins. In contrast, we confirmed that rICL-GFP forms a protein complex with multiple RNA-binding proteins (Eif4g, Fmrp, Pabpc1, Cyfip1 and S6; Fig. 1F). Notably, the GFP-tagged N-terminal domain of ATP1A3 (amino acids M1-P77, NTD-GFP) was still more efficiently expressed in Neuro2a cells than rICL-GFP (Fig. 1C,F). Nonetheless, NTD-GFP did not interact with the translational regulators (Fig. 1F), indicating that RNA-binding proteins specifically interacted with the ICL of ATP1A3 but not with any other overexpressed proteins in Neuro2a cells.

**Fig. 2.**
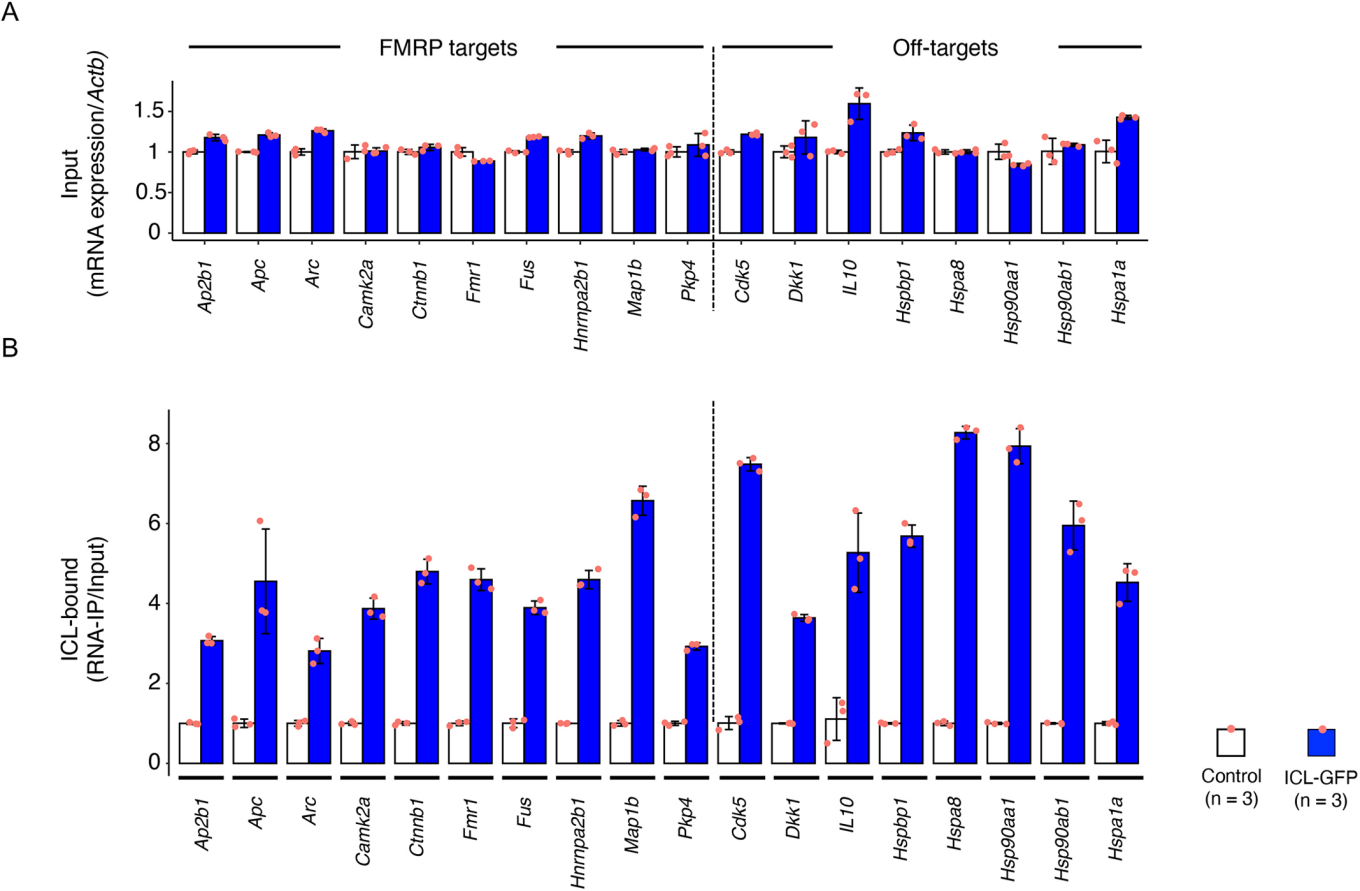
**The intracellular loop of ATP1A3 forms a non-selective ribonucleoprotein complex.** (A) Quantitative PCR for RNAs extracted from Neuro2a cells expressing either GFP (white) or ICL-TET-GFP (blue). (B) Quantitative PCR of the RNA-immunoprecipitated samples. Anti-GFP (clone #RQ2) bead-bound RNAs were subjected to the analysis. For panels A and B, ten genes were selected from the known targets of FMRP for translational regulation, and eight off-target genes were chosen (reference). Data are shown as the mean±s.d. Dots indicate individual data (*n*=3 for each group). *Actb* was used as the internal control. The quantitative results were adjusted for the expression level of each gene (input, panel A). mRNA levels detected by RNA immunoprecipitation were normalized to total mRNA expression levels (RNA-IP/input), and the mean RNA-IP/input value for GFP-bound RNAs was set to 1 (white bars, panel B). Blue bars indicate enrichment of ICL-TET-GFP-bound RNAs.

To demonstrate that ICL-TET-GFP forms a ribonucleoprotein complex, we performed RNA immunoprecipitation (RNA-IP) assays. We chose murine homologs of mRNAs reported to be regulated by FMRP: *Ap2b1*, *Apc*, *Arc*, *CamK2a*, *Ctnnb1*, *Fus*, *Hnrnpa2b1*, *Map1b*, *Pkp4* and *Fmr1* (Pasciuto and Bagni, 2014). The difference in their expression levels remained <50% between cells expressing GFP only and cells expressing ICL-TET-GFP proteins (Fig. 2A). In contrast, the levels of RNAs bound to ICL-TET-GFP were 2.8- to 6.6-fold higher than those bound to controls (GFP), indicating that FMRP-associated RNAs were enriched in the ribonucleotide complex (Fig. 2B). Significant enrichment was also observed in additional RNA-IP assays for ‘off-target’ RNAs of FMRP [*Cdk5*, *Dkk1*, *Il10*, *Hspbp1*, *Hsc70* (also known as *Hspa8*), *Hsp90aa1*, *Hsp90ab1* and *Hspa1a*; Fig. 2B]. The non-selective binding of ICL-TET-GFP to RNAs was consistent with the notion that ICL-TET-GFP forms a complex with the 40S ribosomal subunit S6 and the translational regulators Eif4g and Pabpc1 (Fig. 1F).

**Fig. 3.**
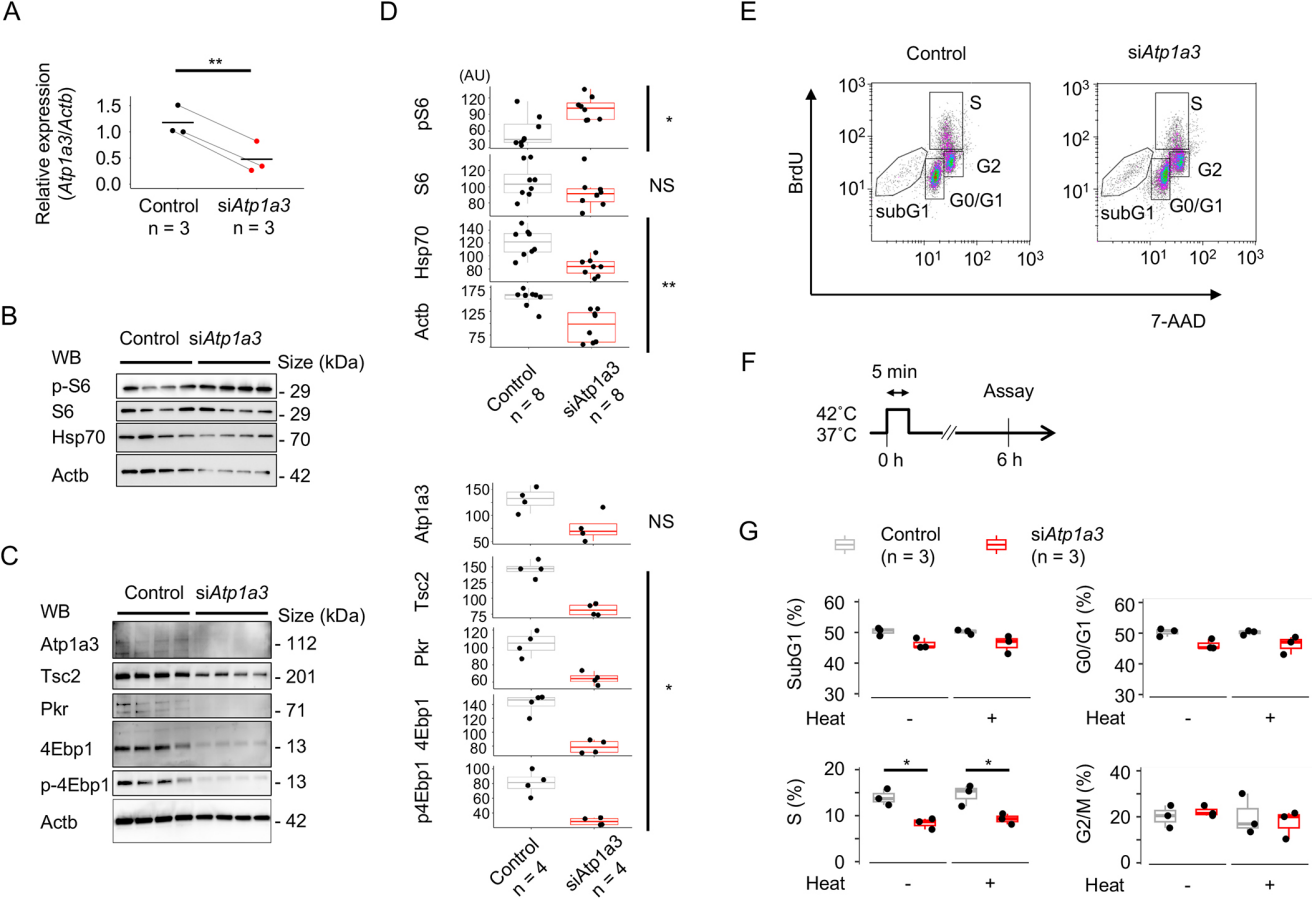
**siRNA-mediated silencing of *Atp1a3* in Neuro2a cells impairs the expression of multiple proteins and cell proliferation.** (A) Relative mRNA expression of *Atp1a3* to *Actb* (internal control) in Neuro2a cells*.* **P<0.01 (three independent experiments, two-tailed paired *t*-test). Bars show mean values. (B,C) Western blotting (WB) for the indicated proteins. Neuro2a cells were treated with control siRNA (lanes 1-4) or si*Atp1a3* (lanes 5-8). Lanes represent four independent replicates. (D) Quantification of protein levels shown in B,C. Dots indicate intensities for individual protein bands in arbitrary units (AU). NS, not significant; *P<0.05; **P<0.01 (control versus si*Atp1a3*; Wilcoxon's rank sum test). (E) Cell cycle analysis by flow cytometry. Panels show representative plots for the fluorescence intensity of 7-AAD (*x*-axis) and FITC-labeled BrdU (*y*-axis). Live cells in the G0/G1, S and G2/M phases (rectangles) and dead cells in the sub-G1 phase (polygons) were gate selected. (F) Schematic of heat treatment (42°C for 5 min) and the time point of the analysis (6 h after heat treatment). (G) Box-dot plots show the percentages of Neuro2a cells in the indicated fractions (sub-G1, G0/G1, S and G2/M phases). The data are summarized for each group with (+) or without (−) heat treatment. *P<0.05 (Tukey's HSD test). Boxes in D,G show the interquartile range, whiskers show the the range of minimum to maximum values, and the median is marked with a line.

These results prompted us to test whether siRNA-mediated depletion of endogenous *Atp1a3* (the murine homolog of *ATP1A3*) causes aberrant molecular signals associated with translational regulation in Neuro2a cells. Quantitative PCR analysis confirmed that the relative expression of *Atp1a3* mRNA in si*Atp1a3*-treated cells was decreased to 40.1% of that in control cells [1.17±0.28 (mean±s.d.) versus 0.470±0.302, *P*=0.0186 (*n*=3 for each group), two-tailed paired *t*-test; Fig. 3A]. The siRNA-treated cells showed significantly higher levels of phosphorylated S6 (p-S6) than control cells (*P*=1.48×10^−3^, Wilcoxon's rank sum test; Fig. 3B-D). Because the extraction buffer only weakly solubilized the endogenous Atp1a3 protein itself, we did not clearly observe the knockdown effect of si*Atp1a3* by western blotting (*P*=0.0571; Fig. 3C,D). By preparing the western blotting samples for Atp1a3 separately from other samples, the knockdown effect of si*Atp1a3* was confirmed at the protein level (Fig. S4). In contrast, the expression and phosphorylation of 4Ebp1 (encoded by *Eif4ebp1*) were significantly decreased in si*Atp1a3*-treated cells (*P*=0.0286 and 0.0286; Fig. 3C,D). Thus, *Atp1a3*-deficient cells appeared to show hyperactive conditions for RNA translation. However, the expression levels of tuberin (Tsc2) and protein kinase R (Pkr, encoded by *Eif2ak2*) decreased to 55.4% and 60.9% (median values) in si*Atp1a3*-treated cells, respectively, compared to those in control cells (*P*=0.0286 and 0.0286, Wilcoxon's rank sum test; Fig. 3C,D). Moreover, Hsp70 and Actb signals were lower in si*Atp1a3*-treated cells than those in controls (median 72.4% and 61.9%; *P*=1.86×10^−3^ and 1.86×10^−3^, respectively; Fig. 3B-D; Fig. S5). It was therefore likely that inefficient RNA translation resulted in a compensatory hyperphosphorylation of ribosomal protein S6 in *Atp1a3*-deficient Neuro2a cells.

### Heat-labile phenotype of *Atp1a3*-deficient cells

As stated above, we observed lower Hsp70 expression levels in *Atp1a3*-deficient cells than in controls (Fig. 3C; Fig. S5). By interacting with various client proteins at different stages of the cell cycle, Hsp70 is known to regulate cell cycle progression (Truman et al., 2012). Thus, we rationalized that *Atp1a3*-deficient Neuro2a cells might show impaired cell cycle progression and cell viability after heat stress (Fig. 3E). In the basal condition, *Atp1a3*-deficient cells showed a lower percentage of cells in S phase in comparison to the percentage of control cells in S phase [‘heat –’: 13.7±1.77% versus 8.69±1.19%, mean±s.d.; *P*=0.0139, Tukey's honestly significant difference (HSD) test; Fig. 3E-G]. No other differences were observed in the resting state. The 5-min heat treatment did not augment the decrease in the S phase population of *Atp1a3*-decifient cells over that seen in the basal condition (‘heat +’: 15.4±2.28% versus 9.3±1.15%; *P*=0.0185; Fig. 3E).

### Mitochondrial dysfunction in *Atp1a3*-deficient cells

**Fig. 4.**
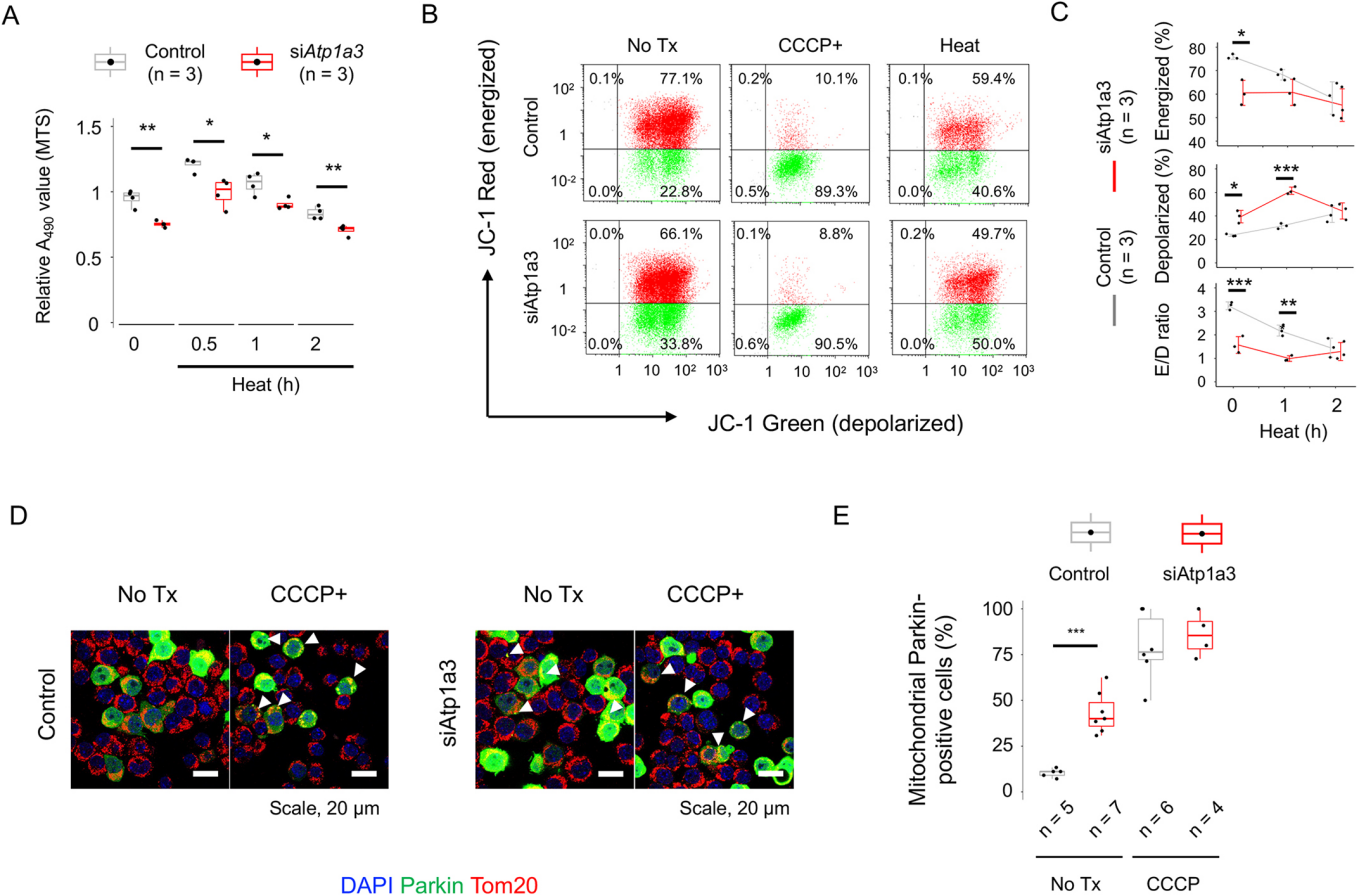
**Susceptibility to heat stress and mitochondrial vulnerability of *Atp1a3*-depleted Neuro2a cells.** (A) MTS assays for control (gray) and si*Atp1a3*-treated (red) Neuro2a cells before and after heat stress (42°C for 0.5-2 h). *P<0.05; **P<0.01 (two-way ANOVA). (B) Flow cytometry analysis of Neuro2a cells with JC-1 staining. The relative populations of energized (red) and depolarized (green) mitochondria are shown. (C) Quantitative JC-1 staining data at each time point after heat treatment. The mean±s.d. values are plotted on a line. *P<0.05; **P<0.01; ***P<0.001 (Tukey's HSD test). (D) Immunofluorescence analysis of control and si*Atp1a3*-treated cells. Arrowheads point to cells with aggregates of Parkin (encoded by *Prkn*) conjugated to EGFP (green) colocalizing with Tom20 (encoded by *Tomm20*, red) signals (red). (E) Quantification of cells positive for mitochondrial parkin localization, as shown in D. ***P<0.001 (Wilcoxon's rank sum test). Boxes in A,E show the interquartile range, whiskers show the range of minimum to maximum values, and the median is marked with a line. In B,D,E: No Tx, no treatment; CCCP+, carbonyl cyanide *m*-chlorophenyl hydrazone treatment; Heat, heat treatment at 42°C for 5 min. Treatments are shown above each panel.

To confirm the heat vulnerability of *Atp1a3*-deficient Neuro2a cells, we incubated the cells at 42°C for 0.5-2 h and compared the number of living cells with those of controls based on the MTS assay. We found that 0.5 h of heat stress caused an increase in MTS activity, which declined to the basal level after 1-2 h of heat stress in both *Atp1a3*-deficient cells and controls (Fig. 4A). At each time point, the MTS activities of *Atp1a3*-deficient cells were lower than those of controls [*P*=8.32×10^−10^ (time) and 3.56×10^−8^ (control versus si*Atp1a3*), two-way ANOVA]. These results support the concept that *Atp1a3*-deficient cells have impaired ATPase and mitochondrial metabolic activity (Rai et al., 2018). The heat-labile phenotype of *Atp1a3*-deficient cells suggests that Atp1a3 exhibits mitochondrial instability. Thus, we analyzed the mitochondrial inner membrane potential (ΔΨm) using the fluorescent indicator 5,5′,6,6′-tetrachloro-1,1′,3,3′-tetraethylbenzimidazolylcarbocyanine iodide (JC-1). Flow cytometry revealed that *Atp1a3*-deficient cells had lower ΔΨm levels [energized/depolarized (E/D) ratio of JC-1] than those of control cells under non-stressed conditions (*P*=0.0317, Tukey's HSD test; Fig. 4B,C). A mitochondrial oxidative phosphorylation uncoupler, 100 µM *m*-chlorophenylhydrazone (CCCP), was used as a positive control. This compound nearly completely abolished the E/D ratio in both control (89.3%) and si*Atp1a3*-treated (90.5%) Neuro2a cells (CCCP+; Fig. 4B). The relative E/D ratios of control cells declined after 60 min (median 67.4%) and 120 min (median 44.9%) of heat treatment in comparison to the ratios before treatment (100%) [*P*=4.13×10^−3^ (control versus si*Atp1a3*) and 7.64×10^−3^ (time), two-way ANOVA; Fig. 4C, lower panel]. Thus, the difference in E/D ratio between control and *Atp1a3*-deficient cells became less evident after 2 h of heat treatment (*P*=0.982) than at 0 h (*P*=1.98×10^−4^) and 1 h (*P*=3.83×10^−3^; Fig. 4C).

We tested whether ouabain, a potent Na^+^/K^+^-ATPase inhibitor, inhibited the ATPase activity of Atp1a3, leading to an increased calcium influx (high Fluo-4 signal) and reduction in mitochondrial membrane potential (high frequency of cells with depolarized JC-1 signal) in Neuro2a cells. When we added 10-100 µM ouabain to the culture medium, we observed an increase in Fluo-4 signals to 116% of those in the mock-treated cells (100 µM ouabain versus control; Fig. S6A,B). In accordance with an increase in the intracellular calcium level, 100 µM ouabain significantly decreased the percentage of cells with depolarized JC-1 signals in comparison with those of the controls [median 6.26% (100 µM ouabain) versus 23.5% (control); *n*=3; *P*<0.001, Dunnett's test; Fig. S6C,D].

The molecular chaperones Hsp70 and Hsp90 are known to protect neurons from degenerative changes by minimizing protein misfolding and maintaining mitochondrial function (Gupta et al., 2020). Therefore, we tested whether *Atp1a3*-deficient cells were prone to mitophagy activation, an organelle-specific machinery of autophagy (Narendra et al., 2008). Under resting conditions, aggregates of parkin (PRKN) tagged to EGFP (EGFP-parkin) were seen more frequently on mitochondria of *Atp1a*-deficient cells than on those of control Neuro2a cells [median 11.1% (control) versus 40.0% (si*Atp1a3*); *P*=5.68×10^−3^, Wilcoxon's rank sum test; Fig. 4D,E]. CCCP robustly increased EGFP-parkin signals in the mitochondria of both control and *Atp1a3*-deficient cells [median 76.4% (control) versus 85.4% (si*Atp1a3*), *P*=0.589, Fig. 4D,E]. These data indicate that *Atp1a3*-deficient cells are vulnerable to mitochondrial stress, but they are capable of activating mitophagy under stressed conditions at an efficiency similar to that in control cells.

These data never exclude the established concept that Na^+^/K^+^-ATPase activities of Atp1a3 play indispensable roles in the maintenance of cell viability, resting and action potential formation in neurons, and their mitochondrial energy production *in vivo* (Arystarkhova et al., 2019; de Lores Arnaiz and Ordieres, 2014; Heinzen et al., 2014; Li et al., 2022). However, the results support an alternative hypothesis that Atp1a3 maintains the mitochondrial membrane potential through Na^+^/K^+^-ATPase-independent mechanisms.

### Dominant effects of the ATP1A3 p.R756C variant on RNA translation

**Fig. 5.**
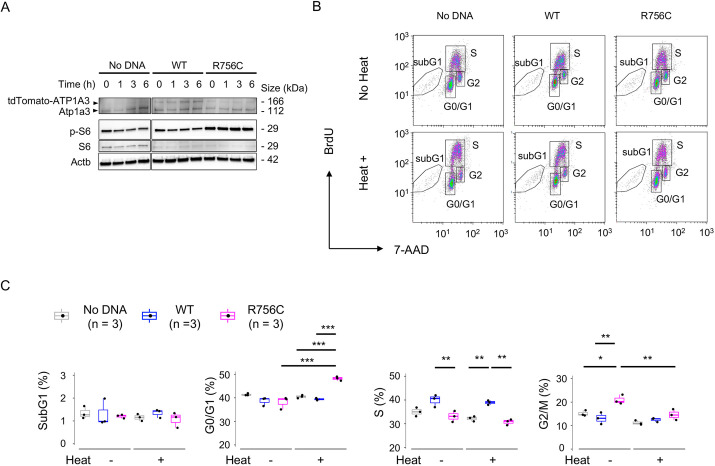
**Heat vulnerability of Neuro2a cells expressing the pathogenic variant p.R756C.** (A) Neuro2a cells were mock transfected (no DNA) or transfected with plasmids expressing tdTomato-tagged wild-type (WT) ATP1A3 or p.R756C variant ATP1A3 and heat treated at 42°C for 5 min. Lysates were collected just before heat treatment (0 h) 1, 3 and 6 h after heat treatment and subjected to western blotting. Both tdTomato-tagged human ATP1A3 and endogenous mouse Atp1a3 were detected using an anti-ATP1A3 antibody. Images are representative of three independent experiments. (B) Cell cycle analysis of Neuro2a cells expressing WT and p.R756C variant-ATP1A3. Gated areas indicate cells in the G0/G1, S and G2/M phases (rectangles) and those in the sub-G1 phase (polygons). (C) Box-dot plots show the percentages of Neuro2a cells in the indicated fractions (sub-G1, G0/G1, S and G2/M phases). The data are summarized for each group with (+) or without (−) heat treatment. Boxes show the interquartile range, whiskers show the range of minimum to maximum values, and the median is marked with a line. *P<0.05; **P<0.01; ***P<0.001 (Tukey's HSD test).

We recently reported a case of recurrent attacks of generalized weakness, involuntary ocular movements and choreoathetosis due to a febrile illness (Kanemasa et al., 2016). As the patient carried a *de novo* p.R756C variant in *ATP1A3*, we investigated whether the AHC (RECA)-associated variant had a dominant effect on RNA translation. To clarify this, we established Neuro2a cells that constitutively expressed wild-type (WT) or the p.R756C mutant of human ATP1A3 as tdTomato fusion proteins (Fig. 5A; Fig. S7). The tdTomato-ATP1A3 (WT) signals clearly showed a membrane-bound pattern, whereas such membrane signals were completely absent in tdTomato-ATP1A3 (R756C)-expressing Neuro2a cells (Fig. S7). Similar to the experiments using siRNA, ATP1A3 (p.R756C)-expressing Neuro2a cells showed higher p-S6 signals both in the basal condition and at 1-6 h after heat treatment in comparison to those in the non-transfected and ATP1A3 (WT)-expressing controls (Fig. 5A).

Consistent with the data from the siRNA experiments (Fig. 3E-G), the overexpression of either WT or p.R756C variant ATP1A3 did not affect the number of cells in the sub-G1 phase before and after heat stress (*P*=0.817, Kruskal–Wallis test; Fig. 5B,C). However, ATP1A3 (p.R756C)-expressing cells showed lower percentages of cells in S phase than ATP1A3 (WT)-expressing cells both before (33.1±2.32% versus 40.6±2.58%; *P*=5.55×10^−3^; Tukey's HSD test) and after (39.0±0.93% versus 31.1±1.22%, *P*=1.15×10^−3^) heat treatment (Fig. 5C, third panel). The expression of ATP1A3 (p.R756C) induced a higher rate of G0/G1 cells after heat treatment in comparison to before the treatment (48.3±0.87% versus 39.4±0.47%, *P*=1.80×10^−5^; ‘G0/G1’, Fig. 5C). In agreement with this change, ATP1A3 (p.R756C)-expressing cells showed a lower rate of G2/M phase after heat treatment in comparison to before the treatment (14.5±2.31% versus 20.2±2.31%, *P*=8.39×10^−3^; ‘G2/M’, Fig. 5C). Thus, we confirmed that the stable expression of WT and p.R756C-variant ATP1A3 had a differential effect on the cell cycle response after heat treatment.

### The pathogenic effects of the p.R756C mutation in patient-derived iPSCs

To further explore the pathogenic effects of the AHC/RECA-related ATP1A3 variant, we established induced pluripotent stem cells (iPSCs) from a healthy individual (control) and a patient with a heterozygous *de novo* p.R756C variant in *ATP1A3* (Fig. S8A,B) (Kanemasa et al., 2016). We verified that ATP1A3 was expressed at comparable levels in the control and patient iPSCs (Fig. S9A,B). Notably, control iPSCs exhibited ATP1A3 (WT) signals distributed homogenously throughout the cytoplasmic region, whereas patient iPSCs showed higher ATP1A3 (p.R756C) signals around the nuclei than in the peripheral cytoplasmic region. We could not detect the membrane-bound pattern of ATP1A3 (WT) signals in iPSCs, which was observed with tdTomato-ATP1A3 (WT) signals in Neuro2a cells (Fig. S7)

**Fig. 6.**
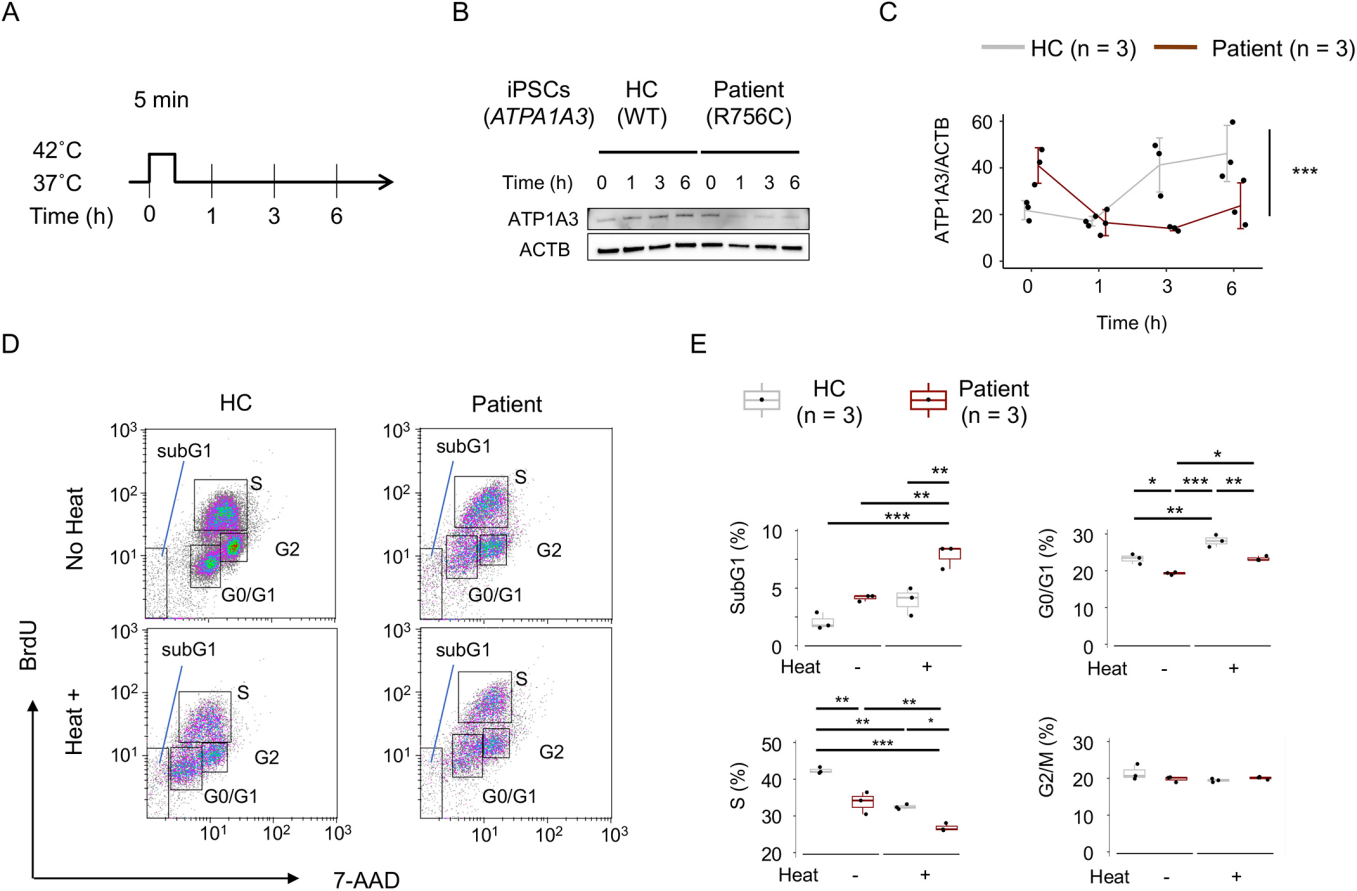
**Susceptibility of patient-derived undifferentiated iPSCs to heat stress.** (A) Schematic of heat stress (42°C for 5 min) and time points of data acquisition (0-6 h) for iPSCs. (B) Western blotting of iPSCs from a healthy control (HC) and a patient with the p.R756C variant in *ATP1A3*. The time points of the analysis are indicated above each lane. (C) Quantification of relative ATP1A3 protein levels over time. Mean±s.d. values are plotted on a line. Individual data are shown as dots. ***P<0.001 (two-way ANOVA). (D) Cell cycle analysis of iPSCs from the HC and patient with the p.R756C variant of *ATP1A3*. Rectangles indicate iPSCs in the sub-G1, G0/G1, S and G2/M phases before (−) and after (+) heat treatment. The incorporated signals of 7AAD (*x*-axis) and FITC-labelled BrdU (*y*-axis) are shown. (E) Box-dot plots show the percentages of Neuro2a cells in the indicated fractions (sub-G1, G0/G1, S and G2/M phases; *n*=3 for each group). The data are summarized for each group with (+) or without (−) heat treatment. Boxes show the interquartile range, whiskers show the range of minimum to maximum values, and the median is marked with a line. *P<0.05; **P<0.01; ***P<0.001 (Tukey's HSD test).

We then compared the susceptibility of the control and patient iPSCs to heat stress (42°C for 5 min; Fig. 6A). Western blotting analysis showed that ATP1A3 expression did not differ between the control and patient iPSCs before treatment (Fig. 6B). In contrast, patient iPSCs expressed Hsp70 at a lower level than did the control iPSCs (Fig. S10). Consistently, heat treatment attenuated the expression of ATP1A3 in patient iPSCs, whereas such a decline did not occur in control iPSCs [*P*=5.86×10^−3^ (time) and 0.0264 (control versus patient), two-way ANOVA; Fig. 6A,B].

Cell cycle analyses characterized patient iPSCs as having a lower percentage of S phase cells than control iPSCs under basal conditions (34.2±3.02% versus 42.0±0.85%; *P*=1.10×10^−3^; Tukey's HSD test; Fig. 6D,E). Heat treatment led patient iPSCs to further suppress proliferating cells, resulting in a lower percentage of S phase cells than that in control iPSCs (26.4±1.07% versus 32.4±0.67%, *P*=0.0150; Fig. 6D,E). Reciprocally, heat treatment induced a higher percentage of sub-G1 phase cells in patient iPSCs than in control iPSCs (8.42±1.02% versus 4.17±1.20%; *P*=2.72×10^−3^; Fig. 6D,E). The difference in the percentage of the sub-G1 phase cells before heat treatment did not reach statistical significance (control, 1.76±0.72%, versus patient, 4.31±0.27%; *P*=0.0769). Thus, we considered that patient iPSCs were more vulnerable to heat stress than control iPSCs.

### Aberrant translation in neurons from the patient-derived iPSCs

To characterize the functional phenotypes of neurons harboring the p.R756C variant of *ATP1A3*, we attempted to differentiate neurons from iPSCs using an established method of serum-free culture of embryoid body (EB)-like aggregates with quick aggregation (SFEBq) (Eiraku et al., 2008) (Fig. S11A). Control iPSCs formed round spheroids that expressed the progenitor markers PAX6, nestin and TUBB3 (Tuj1) after 6 weeks of differentiation (Fig. S11B,C). Patient iPSCs formed neuro-spheroids that were 5-20% smaller in size than control iPSCs during 6 weeks of differentiation (Fig. S12A,B).

**Fig. 7.**
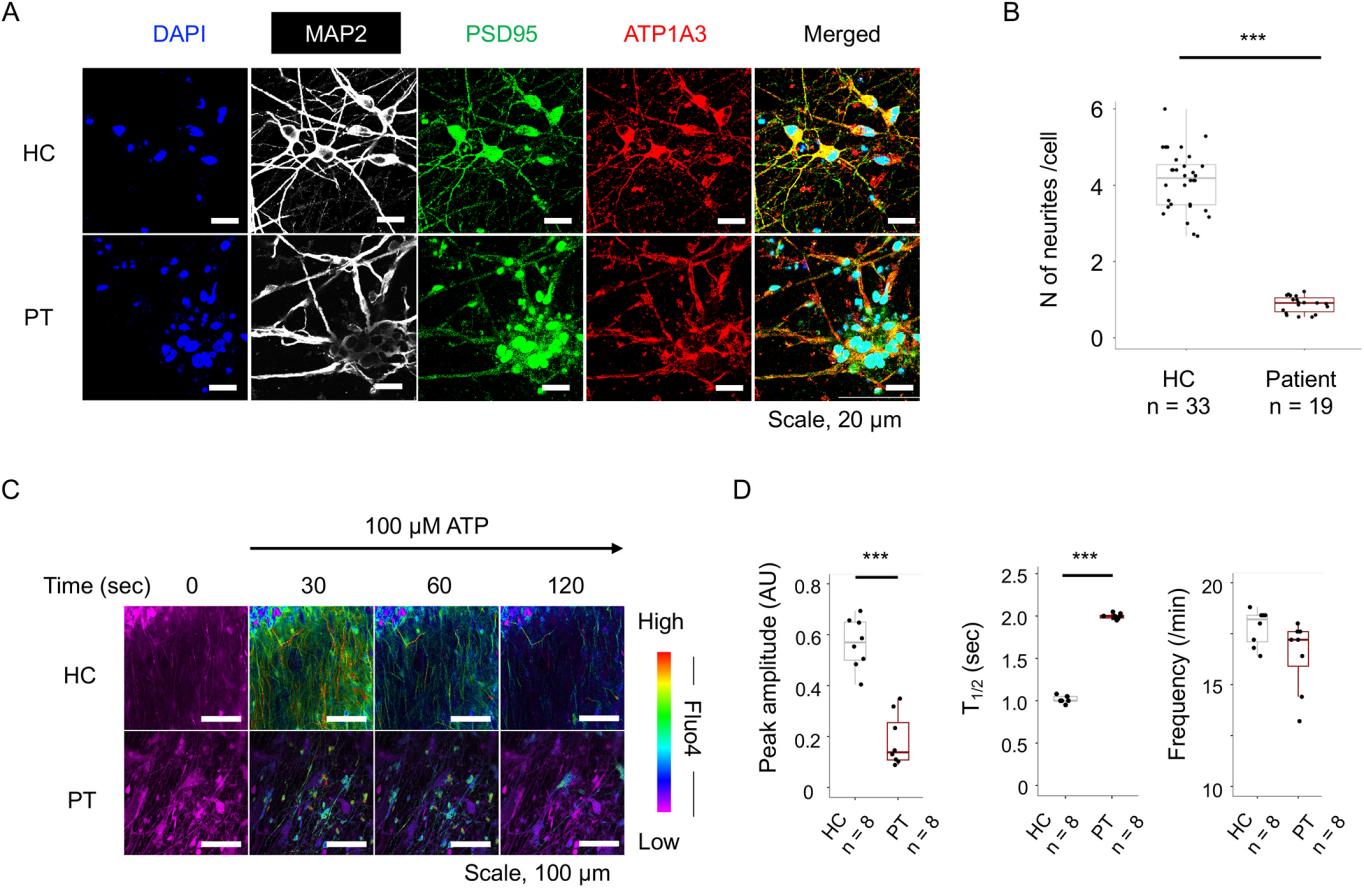
**Morphological and functional analyses of monolayer neurons differentiated from iPSCs.** (A) Immunofluorescence of monolayer neurons from healthy control (HC) and patient (PT) iPSCs with the p.R756C variant in *ATP1A3*. Confocal images show immunolabelled proteins in multicolor channels. Images are representative of three independent experiments. (B) Box-dot plots show the quantitative results for the number of neurites per cell (soma). MAP2 signals were used for the morphological analysis. ***P<0.001 (Wilcoxon's rank sum test). (C) Fluo-4 images before (0 s) and 30-120 s after stimulation with 100 µM ATP of monolayer neurons from HC and PT iPSCs. The color scale indicates low-to-high amplitudes of increased calcium ion levels in neurons. Images are representative of three independent experiments. (D) Quantification of the results shown in C (*n*=8 regions of interest in each group). Box-dot plots show the levels of peak amplitude after stimulation in arbitrary units (AU) (left), duration from peak to 50% Fluo-4 signal (T_1/2_, middle) and oscillating frequency of Fluo-4 signals under basal conditions (right). ***P<0.001 (Wilcoxon's rank sum test). Boxes in B,D show the interquartile range, whiskers show the range of minimum to maximum values, and the median is marked with a line.

Because the ATP1A3 protein is localized in presynaptic terminals and postsynaptic compartments (Liebmann et al., 2013), we analyzed dendritic outgrowth (using MAP2 as a marker) and the expression of a postsynaptic marker (PSD95, encoded by *DLG4*) in neurons from control and patient iPSCs. When we placed neuro-spheroids on iMatrix-511-coated coverslips (#892011, Iwaki Chemicals Co. Ltd.) at 8 weeks of differentiation, neurons began to migrate to the cover glass and extend their neurites radially from the soma (Akamine et al., 2020). Two weeks after plating (8+2 weeks of differentiation), neurons from both the control and patient iPSCs expressed similar levels of ATP1A3, MAP2 and PSD95 (Fig. 7A). However, neurons from patient iPSCs showed fewer dendrites per cell in comparison to neurons from control iPSCs (median 4.19 versus 0.914; *P*=3.30×10^−9^, Wilcoxon's rank sum test; Fig. 7B; Fig. S13).

**Fig. 8.**
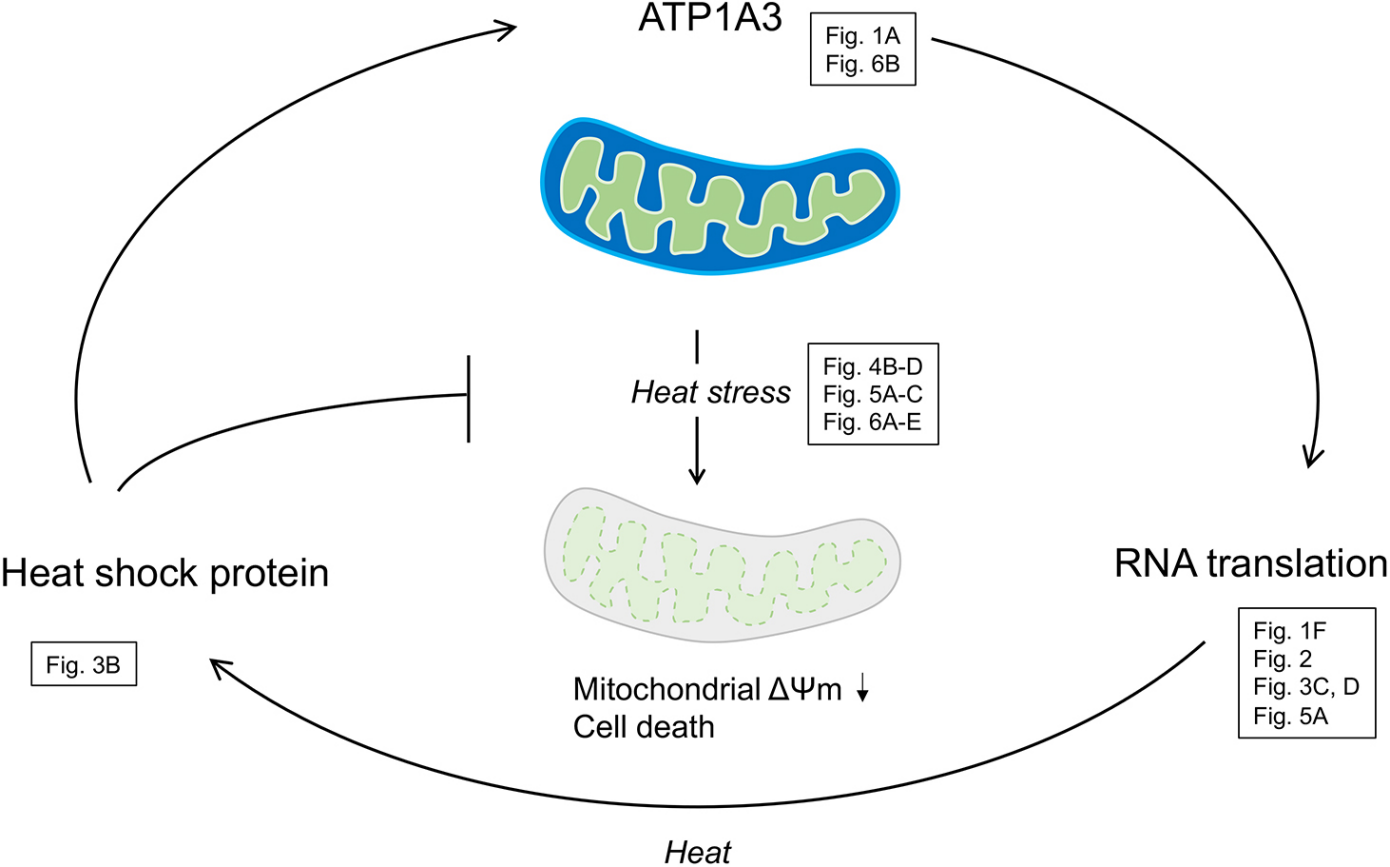
**A theoretical model for ATP1A3-related vulnerability to heat stress.** ATP1A3 supports efficient RNA translation and the expression of heat shock proteins (HSPs) after heat treatment. The pathogenic variant of ATP1A3 (p.R756C) impairs these processes, leading to the restricted expression of Hsp70 and other molecular chaperones. The insufficient expression of HSPs cannot sustain mitochondrial inner membrane potential (ΔΨm) and the expression levels of ATP1A3. The feed-forward loop represents the high-risk condition of patients with *ATP1A3*-related diseases for exaggeration and deterioration under stress conditions.

Finally, we determined whether neurons from patient iPSCs showed functional impairments in response to chemical stimulation with 100 µM ATP (Nascimento et al., 2019). To monitor neuronal excitation, we used Fluo-4, a fluorescent indicator of intracellular calcium ions (Akamine et al., 2020). Monolayer neurons from control and patient iPSCs after 10 weeks of differentiation were subjected to this analysis. We found that iPSCs of patients showed less Fluo-4 signals during the resting condition (0-20 s) than those seen in controls (*P*=1.55×10^−4^, Wilcoxon's rank sum test; Figs S14 and S15). Patient iPSC-derived neurons also showed a lower peak amplitude and a longer duration of excitation (T_1/2_) after stimulation with ATP in comparison to those seen in control neurons [peak amplitude (arbitrary units): median 0.570 versus 0.138, *P*=1.55×10^−4^; duration: median 1.05 s versus 2.01 s, *P*=8.22×10^−4^; Wilcoxon's rank sum test; Fig. 7C,D; Fig. S14). In contrast, control and patient-derived neurons showed similar frequencies in oscillating activity during 3 min of recording (median 18.2 min^−1^ versus 17.2 min^−1^, *P*=0.0806; Fig. 7D). Thus, patient-derived neurons were unable to provoke action potentials after ATP stimulation. Based on these experimental data, we summarize the functional role of ATP1A3 in RNA translation under physiological and stressed conditions (Fig. 8).

## DISCUSSION

We found that the intracellular loop of ATP1A3 interacted with multiple proteins related to post-transcriptional regulation and RNA translation. The knockdown of *Atp1a3* with siRNA and the expression of the ATP1A3 variant p.R756C rendered Neuro2a cells more vulnerable to heat stress than control cells. In agreement with these findings, iPSCs from a patient with the p.R756C variant showed a higher susceptibility to heat stress. Neurons differentiated from patient iPSCs showed fewer dendrites and decreased electroactivity in comparison to those from control iPSCs. Thus, our findings suggest a previously unreported association between RECA, one of the *ATP1A3*-related diseases, and protein synthesis in the differentiating neurons.

Translational regulation is critical for synaptic plasticity and the development of cognitive function in the human brain (Duffy et al., 2022). Among the genes associated with neurodevelopmental disorders, *FMR1* is a well-characterized gene that encodes the RNA-binding protein FMRP. This protein regulates activity-dependent protein synthesis under the control of metabotropic glutamate receptor (mGluR) signaling (Darnell et al., 2011). The molecular pathway downstream of mGluRs involves the assembly of eukaryotic initiation factors and poly A-binding proteins with target mRNAs (polysome complex) for efficient RNA translation and local protein synthesis. As observed in this study, the loss of *Atp1a3* in Neuro2a cells led to the inefficient expression of Hsp70 and instability of the mitochondrial inner membrane potential after heat stress. Given the protein interaction of ATP1A3 (ICL) with Fmrp, Eif4g and Pabpc1 in Neuro2a cells, we speculated that the ICL domain might organize local protein synthesis, which is essential for the development of brain function and resilience to environmental stresses.

From a clinical perspective, one could speculate that there are overlapping molecular mechanisms shared by RECA and other neurodevelopmental disorders, such as tuberous sclerosis, an autosomal dominant genetic disorder characterized by variable degrees of developmental delay, autism spectrum disorder, epileptic seizures and multiple hamartomas in the systemic organs (Curatolo et al., 2022). The disease-responsible genes are *TSC1* and *TSC2*, which encode the two repressor proteins of mTOR signaling, hamartin and tuberin, respectively (Tee et al., 2003). Loss-of-function variations in either *TSC1* or *TSC2* cause hyperactivation of the mTOR pathway, resulting in excessive RNA translation and over-phosphorylation of RPS6 and EIF4E-binding proteins (4EBPs) (Betz and Hall, 2013). In *Atp1a3*-deficient Neuro2a cells, we observed that the expression of TSC2 was lower and the phosphorylation of RPS6 was higher than in control cells. Thus, our data suggest that pathogenic variants in *ATP1A3* lead to the inefficient translation of RNA in neurons, regardless of physiological or stressed conditions, while compensating for such deficits by phosphorylation of RPS6.

More than a decade ago, the Na^+^/K^+^-ATPase in cardiac myocytes was shown to act as a signal transducer (Kometiani et al., 1998). Therefore, it is reasonable to hypothesize that ATP1A3 has versatile functions in addition to its ion transport activity. Ouabain, a classic inhibitor of the Na^+^/K^+^-ATPase, has been widely used for the treatment of heart failure (Schoner and Scheiner-Bobis, 2007); however, the pharmacological effect of ouabain is not limited to blocking the pump function. In fact, it positively or negatively affects cell growth, survival and the expression of genes by modulating various molecular signaling pathways, including the Src and AKT/mTOR pathways (Barwe et al., 2005; Haas et al., 2002). Notably, a recent study showed that ouabain can be used *in vitro* as a neuroprotective agent in an experimental model of Alzheimer’s disease (Song et al., 2019). Because *ATP1A3*-related diseases are known to be exaggerated under various stresses, such as fatigue and infection, it will be valuable to investigate whether the administration of ouabain at an optimal dose could reduce the vulnerability of neuronal activity to heat stress in iPSC-derived neurons or mouse models.

Our data also suggested that the expression of the p.R756C variant of ATP1A3 was unstable in Neuro2a cells and iPSCs exposed to heat stress. This finding is consistent with recent data showing that the p.R756H variant has temperature instability in terms of both protein expression and ATPase activity in cultured cells (Arystarkhova et al., 2023). Besides the heat instability of the p.R756H protein, Snow et al. (2020) reported that both iPSC-derived neurons and knock-in mice expressing the p.E815K protein recapitulated severe phenotypes of patients with an identical variant in *ATP1A3*. The heat-labile phenotypes of p.E815K-expressing neurons and mice were independent of protein expression, suggesting that heat stress disturbed the Na^+^/K^+^-ATPase or still unknown functions of ATP1A3 more profoundly for the p.E815K variant than for the WT protein.

ATP1A3 is a member of the type II P-type ATPase (PAII) protein family, which is highly conserved across mammalian species. Among the dozens of genes encoding PAII family proteins, two more genes are associated with Mendelian diseases: *ATP2A2* and *ATP2C1*, which encode sarcoendoplasmic reticulum Ca^2+^ ATPase (SERCA) and Golgi-localized Ca^2+^/Mn^2+^ ATPase 1, respectively (Vandecaetsbeek et al., 2011). *ATP2A2* is associated with Darier's disease (OMIM #124200), whereas pathogenic variants of *ATP2C1* cause Hailey–Hailey disease (OMIM #169600). Both diseases affect the skin and are clinically exacerbated by elevated ambient temperatures (Szigeti and Kellermayer, 2006). Biochemically, the p.R751Q variant in SERCA showed the highest susceptibility to temperature among multiple pathogenic variants (Kaneko et al., 2014). R751 in SERCA is one of the highly conserved amino acid residues in the PAII family, and the location of R751 in SERCA corresponds to that of R756 in ATP1A3 (Arystarkhova et al., 2023; Toyoshima et al., 2000). Thus, these arginine residues are thought to critically determine their susceptibility to heat stress in terms of both their pump functions and their properties of interaction with other molecules.

HSPs are a group of stress-induced chaperones that facilitate refolding and degradation of abnormal proteins. Thus, loss of the expression or the functional impairment of HSPs is considered an exaggerative factor in neurodegenerative diseases such as Alzheimer's disease, Parkinson's disease and spinocerebellar ataxias (Lackie et al., 2017; Rosenzweig et al., 2019). Consistently, HSPs are known to participate in the process of mitophagy, a selective type of autophagy that eliminates damaged or dysfunctional mitochondria under stress conditions (Yoon et al., 2019). Given the heat vulnerability of the p.R756C variant of ATP1A3, lower translational efficiency may lead to the insufficient expression of Hsp70 and other HSPs in the human brain expressing the pathogenic variant of *ATP1A3*. Consequently, it may further destabilize the mitochondrial inner membrane potential and functional structure of ATP1A3 itself (Fig. 8), mimicking the siRNA-mediated depletion of *Atp1a3* in Neuro2a cells. In this regard, the HSP system can also be a potential therapeutic target for *ATP1A3*-related diseases, as demonstrated in age-associated neurodegenerative diseases (Guo et al., 2023). In addition to heat stress, cold environments are known to induce the expression of HSPs in the mammalian brain (Kaneko and Kibayashi, 2012). The transcriptional activation of HSPs under both kinds of extreme temperatures may reflect the seizure-prone phenotypes of animal models of *ATP1A3*-related diseases under stressed conditions (Helseth et al., 2018; Holm et al., 2016b; Hunanyan et al., 2015). In particular, cold temperatures might become a critical factor for not only exaggerating neurological symptoms, but also provoking autonomic dysfunction and sudden death in patients with *ATP1A3*-related diseases (Kaneko et al., 2014; Kaneko and Kibayashi, 2012; Mikati et al., 2000). From this perspective, it is worth investigating whether iPSC-derived neurons with p.R756C and other variants have impaired expression of HSPs under cold stress. Although Neuro2a cells exhibited tdTomato-ATP1A3 (WT) signals in a membrane-bound pattern, iPSCs did not show such an expression pattern. This difference might be related to the less efficient post-translational modification of ATP1A3 (Arystarkhova et al., 2021) or fewer membrane-bound proteins co-expressed with ATP1A3 in immature cells (iPSCs) than in neuronal cells (Neuro2a) (Smith et al., 2021).

The siRNA-mediated silencing of *Atp1a3* showed milder effects of heat treatment on the cell cycle and viability after heat treatment than the expression of the p.R756C mutant in Neuro2a cells. These observations may explain the more profound effect of missense variants than haploinsufficiency on the clinical presentation of *ATP1A3*-related disorders (Clapcote et al., 2009; Helseth et al., 2018; Holm et al., 2016b; Hunanyan et al., 2015, 2018; Ikeda et al., 2013). However, we cannot exclude the possibility that patients with the p.R756C variant are more vulnerable to heat stress than those with truncating mutations, irrespective of functional deficits in RNA translation or mitochondrial stability in neurons. Microelectrode arrays and more quantitative methods than the current experimental system must be established to analyze the degree of deleterious effects of haploinsufficiency compared to those of missense variations (Snow et al., 2020). Pharmacological interventions with activators of ribosomal (Kusnadi et al., 2020) and mitochondrial biogenesis (Zheng et al., 2023) may further delineate the pathogenic effects of RNA translation and mitochondrial instability.

The present study was associated with some limitations. First, we did not clarify which step of ATP1A3 is regulated in the complex reaction system of cap-dependent translation. The cell-free reconstitution system of *in vitro* translation with and without the purified ATP1A3 protein may provide clues to answer this question. Second, we did not investigate whether ATP1A3 was required for protein synthesis *in vivo*. It also remains to be determined whether neurons regain normal calcium influx and mitochondrial stability with recovery of RNA translation after pharmacological interventions *in vitro.* The activity-dependent translation is the basic mechanism underlying synaptic plasticity. Because each type of brain tissue is thought to develop unique neural circuits, the expression of distinctive protein subsets may be required for their developmental processes. From this perspective, the biochemical interaction of ATP1A3 with the translational machinery may explain the variable effects of a pathogenic variant on each type of tissue in the developing brain. The differential effects of translation may also be applied to phenotypic divergence among individuals with *ATP1A3* variations. We cannot still conclude that the increased pS6 level in p.R756C-expressing iPSCs reflects the condition of hyperactive RNA translation under the resting and stressed conditions. Contrarily, it might be reasonable to hypothesize that p.R756C-expressing neurons try to compensate for inefficient RNA translation. From this perspective, it is unlikely that they can accelerate the speed of protein synthesis under stressed conditions, even when a higher amount of HSPs is required than in the resting condition. Third, we did not perform a transcriptome-wide analysis of ATP1A3-bound RNAs. Delineating the differential subsets of RNAs bound to WT and variant ATP1A3 will help us better understand the molecular pathways associated with the exaggerated process. Lastly, the absence of an isogenic control in patient-derived iPSCs has left the genotype-phenotype correlation unresolved. CRISPR/Cas9-mediated conversion of p.R756C to wild-type ATP1A3 may confirm the cause-and-effect relationship between variant protein expression and RNA translation/mitochondrial instability in iPSC-derived neurons.

In conclusion, this is the first study to develop a protein interaction network for ATP1A3. Using this approach, we hypothesize that an *ATP1A3*-related syndrome (RECA) is closely linked to the molecular mechanisms of RNA translation and heat shock response. Using brain organoids from iPSCs and mouse models, our data will provide further insight into the molecular targets of therapeutic interventions in future translational research.

## MATERIALS AND METHODS

### Plasmids

Total RNA was extracted from peripheral blood mononuclear cells (PBMCs) of a healthy volunteer using an RNeasy Kit (#74106, QIAGEN, Valencia, CA, USA). Complementary DNA (cDNA) was synthesized using a High-Capacity RNA-to-cDNA kit (#4387406, Thermo Fisher Scientific, Waltham, MA, USA), according to the manufacturer's protocol. Partial fragments encoding T335 to L839 of ATP1A3 and full-length cDNAs were amplified using Platinum SuperFi DNA Polymerase (#12351-010, Thermo Fisher Scientific) (Table S2). Amplicons were cloned into the pEGFP-N2 or ptdTomato-N1 vector (#632532, Takara Bio, Shiga, Japan) using an In-Fusion HD Cloning Kit (#639648, Takara Bio). The original ICL-GFP construct was prepared according to the domain information in the Human Protein Reference Database, which was no longer available as of November 2023. The updated information from the UniProt database (https://www.uniprot.org/uniprotkb/P13637/entry) revealed that the T335 to L839 fragment involved one extracellular domain (F783-L792), two transmembrane domains (K763-L782 and G793-A813) and the ICL III domain (Y814-K833) adjacent to the ICL II domain (T329-L762) (Fig. 1A). Thus, we designed two additional constructs expressing the N-terminal domain (NTD: M1-P77) and refined ICL II (rICL: T329-L762) fused to GFP (Fig. 1A,B). NTD served as a reference for the structurally stable, ICL-irrelevant region in ATP1A3 (Sweadner et al., 2019). p.R756C and other mutations were introduced using QuikChange II XL Site-Directed Mutagenesis Kits (#200521, Agilent Technologies, Wilmington, DE, USA). Plasmid pEGFP-parkin WT (encoding human PRKN congujated to EGFP) was purchased from Addgene (Table S2).

### Establishment of stable cell lines

Neuro2a cells were cultured in Dulbecco's Minimal Essential Medium (Wako, Tokyo, Japan) containing 10% heat-inactivated fetal calf serum (#10270106, Gibco) and 1% penicillin/streptomycin (Nacalai Tesque, Kyoto, Japan) at 37°C in a normoxic environment with 5% CO_2_ and 100% humidity.

Plasmid transfection was performed using Lipofectamine 3000 (Thermo Fisher Scientific). EGFP- or tdTomato-expressing cells were selected three times over a period of 4 weeks using a Cell Sorter SH800 (Sony, Tokyo, Japan) until more than 95% of cells showed GFP or tdTomato fluorescence.

### Knockdown of *ATP1A3*

Transfection with small interfering RNA (siRNA) was performed using Lipofectamine RNAiMAX (Thermo Fisher Scientific). Pre-designed Stealth siRNA against *ATP1A3* (#HSS100796, Thermo Fisher Scientific) and a negative control (#12935300, Thermo Fisher Scientific) were used (Table S2).

### Co-IP

Neuro2a cells stably expressing GFP or ICL-TET-GFP were used for the co-IP-based protein screening. A total of 1×10^8^ cells were harvested at the exponential growth phase at 70-80% confluence in four to five 100 mm dishes (Akamine et al., 2020). Cells were homogenized in ice-cold lysis buffer (0.5% Triton X-100, 150 mM NaCl, 20 mM Tris-HCl, pH 7.5 and 2 mM EDTA) supplemented with proteinase inhibitor (#11873580001, Roche, Rotkreuz, Switzerland) and phosphatase inhibitor cocktails (#04906837001, Roche). Cell lysates were pre-cleared with 30 µl of Protein G Sepharose 4 Fast Flow (GE Healthcare, Chicago, IL, USA). Total protein in the supernatants was measured using a Qubit 2.0 fluorometer (Thermo Fisher Scientific) and was adjusted to 0.5 mg/ml with lysis buffer. Immunoprecipitation was performed in 1000 µl lysis buffer containing 50 µl of slurry of anti-GFP monoclonal antibody-conjugated magnetic beads (#D153-11, MBL, Woburn, MA, USA) and 1% bovine hemoglobin (Sigma-Aldrich, St. Louis, MO, USA). The samples were incubated overnight at 4°C. Beads were washed four times and bead-captured proteins were eluted with Laemmli sampling buffer (Bio-Rad, Hercules, CA, USA) containing 5% 2-mercaptoethanol. After boiling at 95°C for 5 min, the eluted proteins were separated on 4-15% gradient precast gels (#64512262, Bio-Rad). Proteins were visualized using Coomassie Brilliant Blue (#11642-31, Nacalai Tesque). The detected bands were diced into small pieces (1-2 mm squares) for mass spectrometry.

### RNA-IP

Cleared lysates were prepared as described above and supplemented with an RNase inhibitor (#N2611, Promega, Madison, WI, USA) in lysis/immunoprecipitation buffer. After blocking the anti-GFP beads (MBL) with 100 µg/ml yeast tRNA (#AM7119, Invitrogen, Waltham, MA, USA), immunoprecipitation was performed for 1 h at 4°C. The beads were washed four times, and protein-bound RNAs were recovered using the RNeasy Micro Kit (QIAGEN). Quantitative PCR was performed using SYBR Green (#4385612, Thermo Fisher Scientific).

### Liquid chromatography with tandem mass spectrometry

Diced electrophoresis gels of the protein bands were subjected to in-gel digestion as previously described (Shevchenko et al., 2006). Trypsin-digested peptides were separated on an Easy-nLC1000 system (Thermo Fisher Scientific) using an Acclaim PepMap 100 trap (#164946; 20×0.075 mm, 3 μm; Thermo Fisher Scientific) and Acclaim PepMap RSCL analytical columns (#164943; 150×0.05 mm, 2 μm; Thermo Fisher Scientific). A Q-Exactive Orbitrap mass analyzer (Thermo Fisher Scientific) was used as the mass spectrometry detector. Proteins were identified and quantified using the Proteome Discoverer software program (Thermo Fisher Scientific) using the search algorithm Sequest HT against the UniProt database (The UniProt, 2017).

### Cell cycle analysis

After heat treatment, cell cycle analysis was performed using the Bromodeoxyuridine (BrdU) Flow kit (BD Pharmingen, San Diego, CA, USA) according to the manufacturer's protocol (Ohkubo et al., 2015). Briefly, 10 μM BrdU was added to the culture medium for 30 min, and cells were trypsinized, fixed and labeled with FITC-labelled anti-BrdU monoclonal antibody and 7-amino-actinomycin D (7-AAD). An EC800 Flow Cytometry Analyzer (version 1.3.6, SONY Biotechnology, Tokyo, Japan) was used to measure fluorescence. The acquired data were visualized using the Kaluza software program (Beckman Coulter).

### Mitochondrial membrane potential

The MitoProbe JC-1 Assay Kit (#M34152, Thermo Fisher Scientific) was used for measurement of mitochondrial inner membrane potential (Yonemoto et al., 2023). In brief, Neuro2a cells were treated with 200 nM control siRNA, 200 nM si*Atp1a3* or 10-100 µM ouabain (#O3125; Sigma-Aldrich) for 48 h before the JC-1 assay. Cyanide *m*-chlorophenyl hydrazone (CCCP; available in the JC-1 Assay Kit, Thermo Fisher Scientific) was added to the culture medium (100 µM, 37°C for 5 min) to disrupt mitochondrial oxidative phosphorylation. Cells were dissociated with trypsin, collected and resuspended in 37°C phosphate-buffered saline (pH 7.4). JC-1 signals were analyzed using flow cytometry. Cells emitting red fluorescence of polymerized JC-1 were defined as harboring energized mitochondria, whereas those with green signals (JC-1 monomer) were characterized as having depolarized mitochondria.

### Quantitative real-time PCR

First-strand cDNA was synthesized using a High-Capacity RNA-to-cDNA Kit (Thermo Fisher Scientific). Quantitative real-time PCR was performed using Fast SYBR Green Master Mix (Thermo Fisher Scientific) and the StepOnePlus Real-Time PCR System (Thermo Fisher Scientific). Murine β-actin (*Actb*) was used as an internal control (Table S2). The PCR conditions were 95°C (20 s), followed by 40 cycles of 95°C (3 s) and 60°C (30 s). The relative gene expression was calculated using the ddCt method (Akamine et al., 2020).

### MTS assay

To analyze cell proliferation, we carried out MTS [3-(4,5-dimethylthiazol-2-yl)-5-(3-carboxymethoxyphenyl)-2-(4-sulfophenyl)-2H-tetrazolium] assays using the CellTiter 96 AQueous One Solution Cell Proliferation Assay System (#G3582; Promega, Madison, WI, USA). Prior to transfection with siRNA, Neuro2a cells were seeded in 96-well culture plates at 5×10^3^ cells per well. Cells were maintained in DMEM supplemented with 10% FBS and 1% penicillin/streptomycin (100 μl per well) at 5% CO2 and 37°C for 24 hours. For the MTS assay, culture medium in each well was replaced with MTS solution at 4:1 (v:v). Assay plates were incubated at 37°C for 1 h, and absorbance at 490 nm was determined with the Tecan Multiskan GO spectrophotometer (Thermo Fisher Scientific).

### Western blotting

Exponentially growing cells were heat stimulated at 42°C for 5 min and immediately returned to normal conditions at 37°C with 5% CO_2_. Whole-cell lysates were prepared 1, 3 and 6 h after heat treatment using Laemmli buffer (Bio-Rad). The standard protocol for western blotting was used (Akamine et al., 2020). Briefly, the separated proteins were transferred to a polyvinylidene difluoride membrane using a Trans-Blot Turbo Transfer System (Bio-Rad). After blocking with 5% skim milk, the membranes were incubated with primary antibodies at 4°C overnight. The membranes were then washed three times for 10 min with PBS containing 0.5% Tween 20 and incubated with a 1:5000 dilution of horseradish peroxidase-conjugated anti-mouse (#115-035-174) or anti-rabbit secondary antibodies (#211-032-171, Jackson Immunoresearch, West Grove, PA, USA) for 1 h (see Table S2). Chemiluminescence signals were detected using the FluorChem FC2 system (ProteinSimple, San Jose, CA, USA). ACTB (or murine Actb) was used as the internal control. The primary antibodies are listed in Table S2.

### Immunofluorescence

The cells were washed twice with PBS, fixed in 4% paraformaldehyde for 15 min, and permeabilized with 0.3% Triton X-100 for 10 min (Matsushita et al., 2016). After blocking with Block Ace (KAC, Kyoto, Japan) for 30 min, the cells were incubated overnight with primary antibodies at 4°C (Table S2). Alexa Fluor 488-, 555- and 647-conjugated secondary antibodies (1:1000 dilution, Thermo Fisher Scientific) were used for fluorescence labeling, and 4′,6-diamidino-2-phenylindole (DAPI, Thermo Fisher Scientific) was used for nuclear staining. Confocal images were obtained using an A1 HD25 microscope (Nikon, Tokyo, Japan). A BZ-X800 microscope equipped with BZ-X software (Keyence, Osaka, USA) was used for quantitative analysis. Mitochondrial localization of EGFP-parkin was assessed by immunolabelling with anti-GFP antibody (Table S2).

### Calcium imaging

Fluo-4 AM (#CS22, Dojindo, Kumamoto, Japan) was used for calcium imaging according to the manufacturer's protocol. Briefly, the cells were loaded with 10 µM Fluo-4 acetoxymethyl ester calcium indicator at 37°C for 60 min. The loading buffer was then replaced with the reaction buffer for Fluo-4 imaging. Cells were imaged every 1 s under an A1 HD25 microscope (Nikon) equipped with GFP filters and stimulated with 100 µM ATP for 30 s. Signal intensity in the region of interest was measured using NIS-Elements AR software (Nikon).

### iPSCs

PBMCs were isolated by density gradient centrifugation using Lymphocyte Separation Medium (#50494X, MP Biomedical, CA, USA). PBMCs were stimulated with Dynabeads Human T-Activator CD3/CD28 (Thermo Fisher Scientific) in the KBM502 medium (Kohjin Bio, Saitama, Japan). On day 6 of stimulation, activated T cells were isolated using a magnet and infected with Sendai virus vectors expressing four Yamanaka factors: Oct4, Sox2, c-Myc and Klf4 (CytoTune-iPS 2.0 Sendai Reprogramming Kit; DNAVEC, Tokyo, Japan) (Akamine et al., 2020). The medium was changed on day 1 to remove the Sendai virus vector. Cells were plated onto mouse embryonic fibroblast feeder cells on day 3 and fed in Primate ES Cell Medium (#RCHEND001, ReproCELL, Tokyo, Japan) supplemented with bFGF (#RCHEOT002, ReproCELL) until colonies formed. Colonies of iPSCs were manually picked 3-4 weeks after infection. The iPSCs were adapted to feeder-free conditions and maintained on Geltrex-coated plates (#A14133, Thermo Fisher Scientific) in StemFlex medium (#A3349401, Thermo Fisher Scientific) according to the manufacturer's instructions. All iPSCs were cultured at 37°C in a normoxic environment under 5% CO_2_ with 100% humidity.

The obtained iPSCs were tested for the expression of stem cell-specific markers, NANOG, OCT4, TRA-1-60, SSEA-3 and alkaline phosphatase using the ES/iPS Cell Characterization Kit (SAB-KIT-1, System Biosciences, Palo Alto, CA, USA). The differentiation potential of iPSCs was determined by EB formation. Two weeks after the formation of the EB, cells were stained with antibodies against the ectodermal marker TUBB3 (Tuj1 antibody), the mesodermal marker α-SMA and the endodermal marker AFP (#A25538, 3-Germ Layer Immunocytochemistry Kit; Thermo Fisher Scientific). Complete elimination of the Sendai virus was confirmed using the iPS Transgene/SeV detection primer kit (#IDT-DV0301, MBL). Genomic DNA was tested using the hPSC Genetic Analysis Kit (#07550, Stem Cell Technologies, Vancouver, Canada), which detects frequent karyotypic abnormalities in human iPSCs. Undifferentiated iPSCs were used to characterize their heat lability and expression profiles of ATP1A3 (WT and p.R756C).

### Neuronal differentiation

Neuronal differentiation of iPSCs was induced by the SFEBq method (Eiraku et al., 2008). In brief, human iPSCs were treated with 10 mM Y-27632 (#257-00511, Wako, Osaka, Japan) and dissociated into single cells using TrypLE Express solution (#12605-010, Thermo Fisher Scientific). The 1×10^6^ iPSCs were suspended in 15 ml of 5% DFK medium [DMEM/Nutrient Mixture F-12 Ham (#D8437, Sigma Aldrich), 5% Knockout Serum Replacement (#10828010, Thermo Fisher Scientific), 100 mM nonessential amino acids solution (#11140050, Thermo Fisher Scientific), 2 mM L-glutamine and 110 mM 2-mercaptoethanol, supplemented with 10 mM Y-27632, 2 mM dorsomorphin (#0443371, Wako) and 10 mM SB431542 (#192-16541, Wako)]. Suspended iPSCs (150 μl, 1×10^4^ cells) were transferred to a low-attachment 96-well U-bottom plate (#174925, Thermo Fisher Scientific) and then centrifuged at 200 ***g*** for 1 min. Half of the medium in the wells was replaced with 5% DFK medium every 4 days. After 14 days of culture, the expression of neural progenitor cell markers (nestin and PAX6) and a neuronal marker (MAP2) in EBs were confirmed by immunofluorescence analysis. The remaining EBs were transferred to a low-attachment 96-well flat-bottomed plate (Corning, Medfield, MA, USA) and cultured in neurobasal medium (#21103049, Gibco) supplemented with 2% B27 supplement (#17504044, Gibco) and 2 mM L-glutamine. After 90-100 days of culture, neural organoids were plated on 35-mm multi-well glass-bottomed dishes (#D141400, Matsunami Glass, Osaka, Japan) coated with iMatrix 511 (#892012, Takara Bio) for immunofluorescence studies and calcium imaging of neurons.

### Bioinformatics

All statistical analyses were performed using R version 4.3.1 (https://R-project.org) and the JMP software program (version 16, SAS Institute, Cary, NC, USA). The Database for Annotation, Visualization and Integrated Discovery (DAVID; https://david.ncifcrf.gov/) was used for the GO and pathway analyses of the proteins identified in the co-IP study. A network analysis was performed using the STRING database (https://string-db.org/).

### Statistical analysis

The data are shown as the mean±s.d. unless otherwise stated. Wilcoxon's rank sum test was used to compare continuous variables. χ^2^-test was applied to test differences in categorical variables. Statistical significance was set at *P*<0.05.

### Ethics

All experimental procedures were performed in strict compliance with the institutional guidelines and protocols approved by the Institutional Review Board of Kyushu University for clinical studies and experiments with human samples (#28-88, #29-393 and #678-01). The iPSCs were established after obtaining consent from a healthy donor and the patient's parents.

## Supplementary Material

10.1242/dmm.050574_sup1 Supplementary information
