# Supplementary material for: ATP1A3 regulates protein synthesis for mitochondrial stability under heat stress
Source: Dis Model Mech. 2024 Jul 2;17(6):dmm050574. doi: 10.1242/dmm.050574 (PMC11247502; doi:10.1242/dmm.050574)
Supplement: Supplementary information [file dmm-17-050574-s1.pdf]

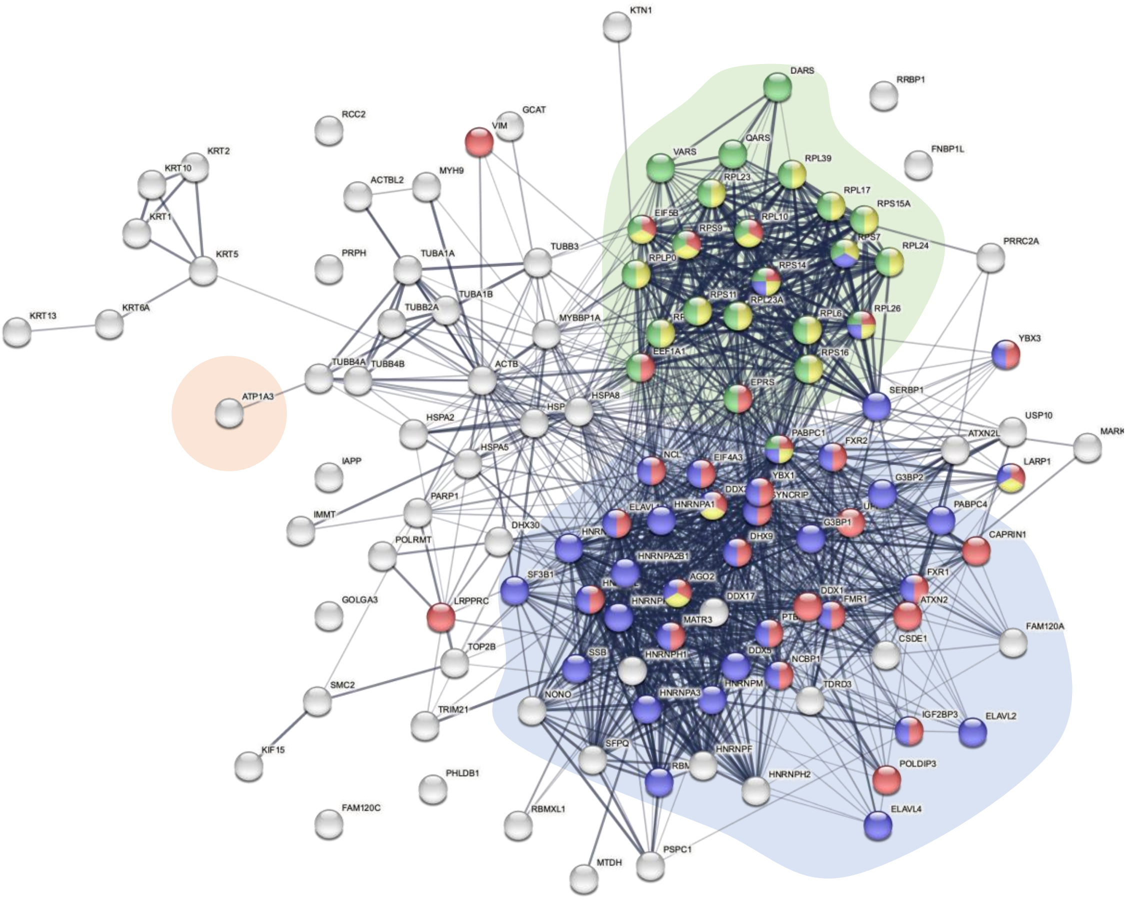

Fig. S1. The interaction network for ATP1A3-binding proteins.

The ATP1A3 (ICL-TET)-binding proteins are fully annotated with symbols in this display. Same color codes are used to indicate the protein functions: Translational initiation (yellow), Translation (green), Regulation of translation (red), and mRNA binding (blue). Color shades indicate the presence of two clusters in the network. Proteomic screening used GFP-fused fragments containing intracellular loops (ICL) II and III, transmembrane domains (T), and extracellular domains (E) of ATP1A3.

Disease Models & Mechanisms • Supplementary information

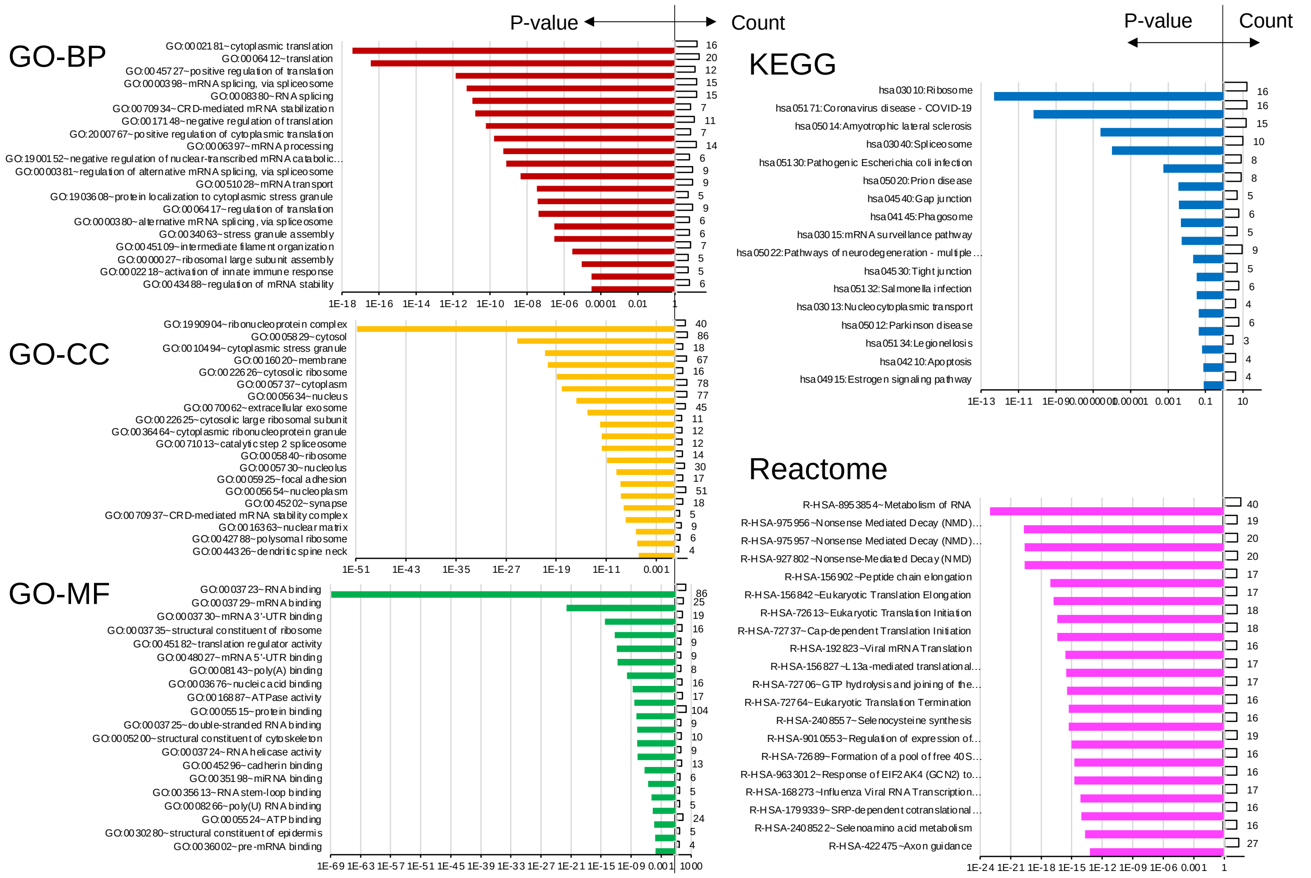

Fig. S2. Summary of bioinformatics analysis.

Enrichment of genes in the specific categories of Gene ontology (GO)-biological process (BP), cellular component (CC), molecular function (MF), Kyoto Encyclopedia for Gene and Genomes (KEGG), and Reactome databases. Bar graphs show the p-values and the number of genes classified into each category.

Disease Models & Mechanisms • Supplementary information

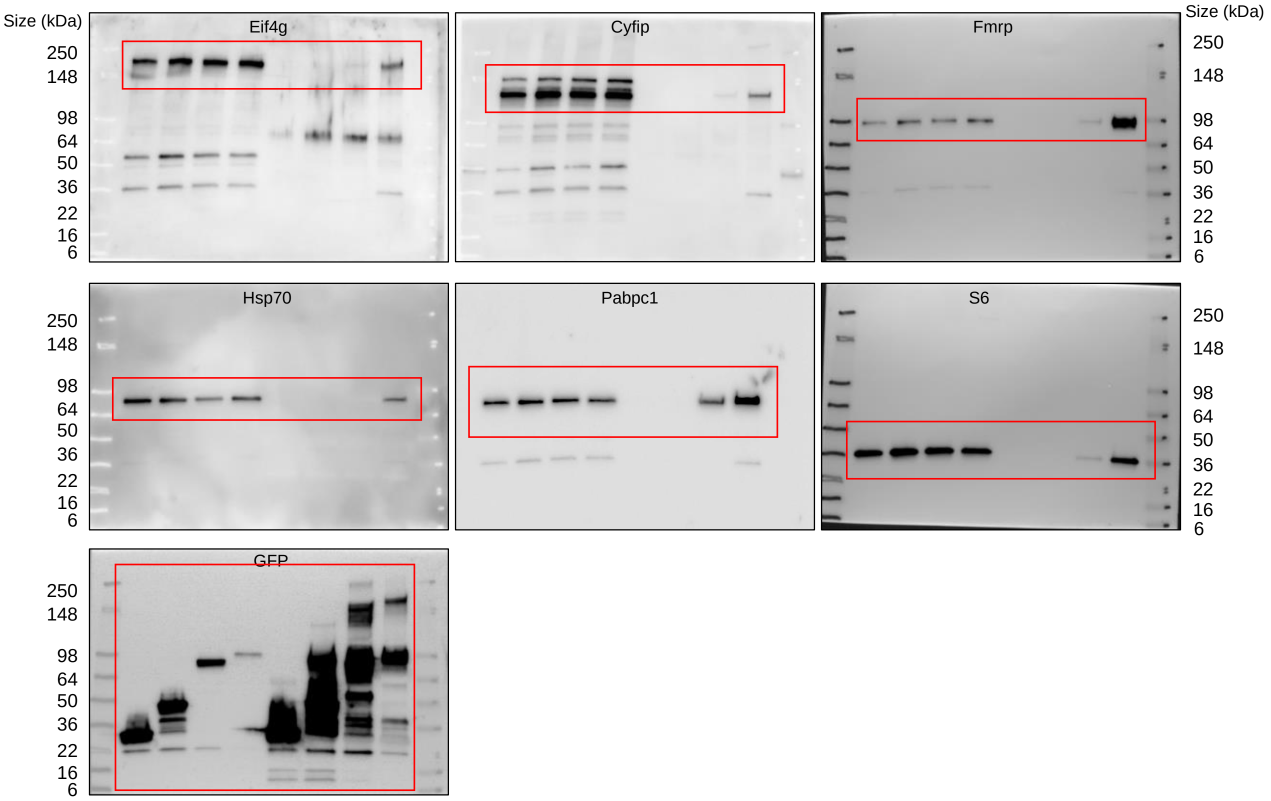

Fig. S3. The full-membrane images of Western blotting.

Chemiluminescent signals are shown as full-membrane images. Red squares indicate the trimmed areas of the main panels in **Fig. 1F**.

Disease Models & Mechanisms • Supplementary information

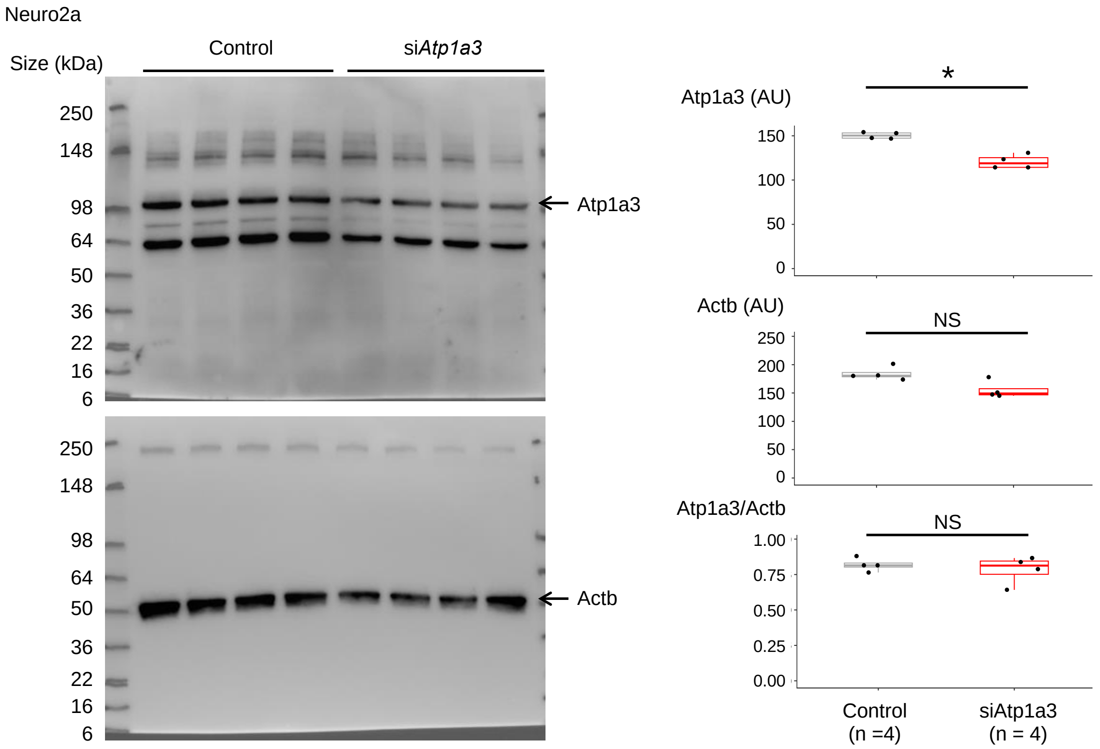

Fig. S4. Atp1a3 signals in Neuro2a cells with or without siRNA treatment.
Western blotting shows endogenous Atp1a3 signals at 100 kDa in control Neuro2a cells (controls, lanes #1 – 4), which decreased to a median of 80.0% after treatment with Atp1a3-specific siRNA (siAtp1a3, lanes #5 – 8). The samples were subjected to SDS-PAGE without boiling. All other samples used for Western blotting were boiled at 99 °C for 2 min. Right: Box-dot plots showing the quantitative measurement of Atp1a3, Actb, and the relative signals of Atp1a3/Actb (controls vs. siAtp1a3). *$p<0.05$ (Wilcoxon's rank-sum test, n = 4 for each). AU, arbitrary unit. NS, not sig50 nificant.

Disease Models & Mechanisms • Supplementary information

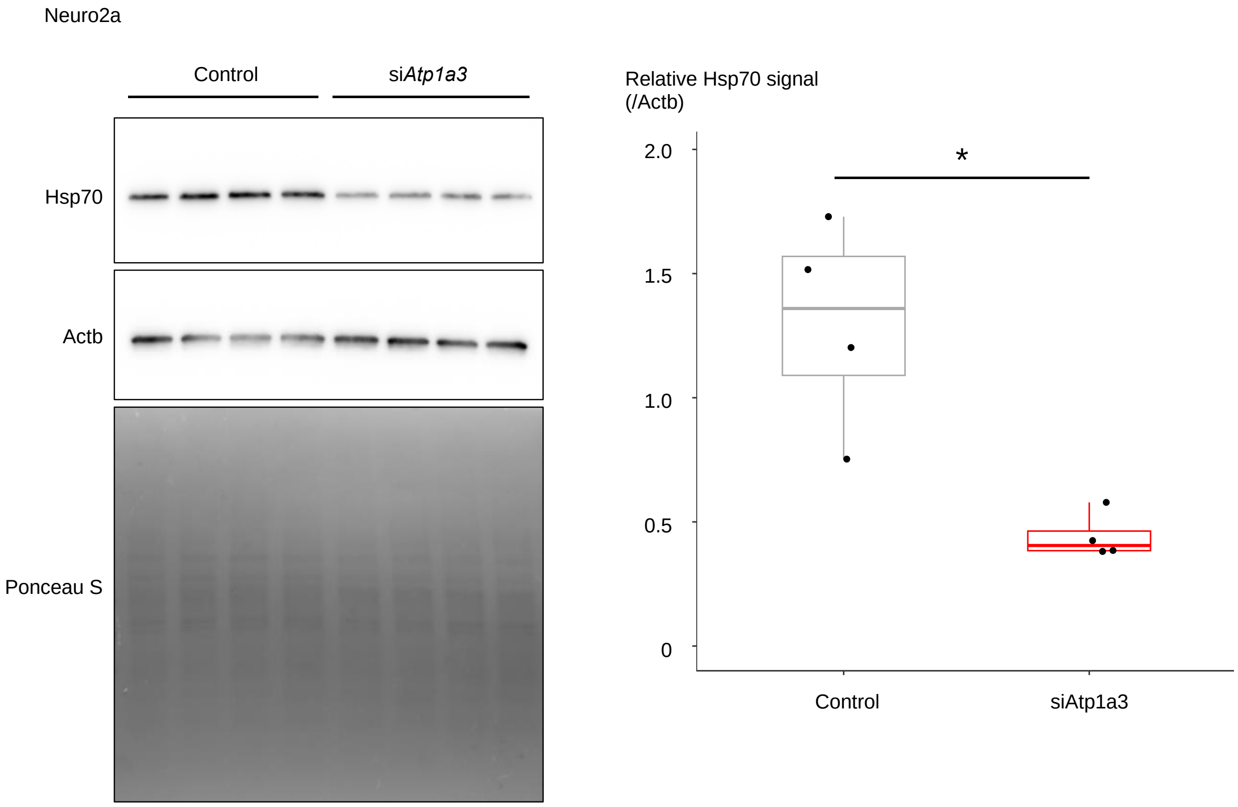

Fig. S5. Hsp70 expression in siAtp1a3-treated and control Neuro2a cells.

Western blotting (left) shows lower expression of Hsp70 in Neuro2a cells treated with siRNA against Atp1a3 (siAtp1a3) than in control Neuro2a cells. Ponceau-S was used to visualize the amount of total protein loaded in each lane. Box-dot plots (right) show the quantitative measurement of Hsp70 signals relative to Actb. *$p < 0.05$ (Wilcoxon's rank-sum test, n = 4 each).

Disease Models & Mechanisms • Supplementary information

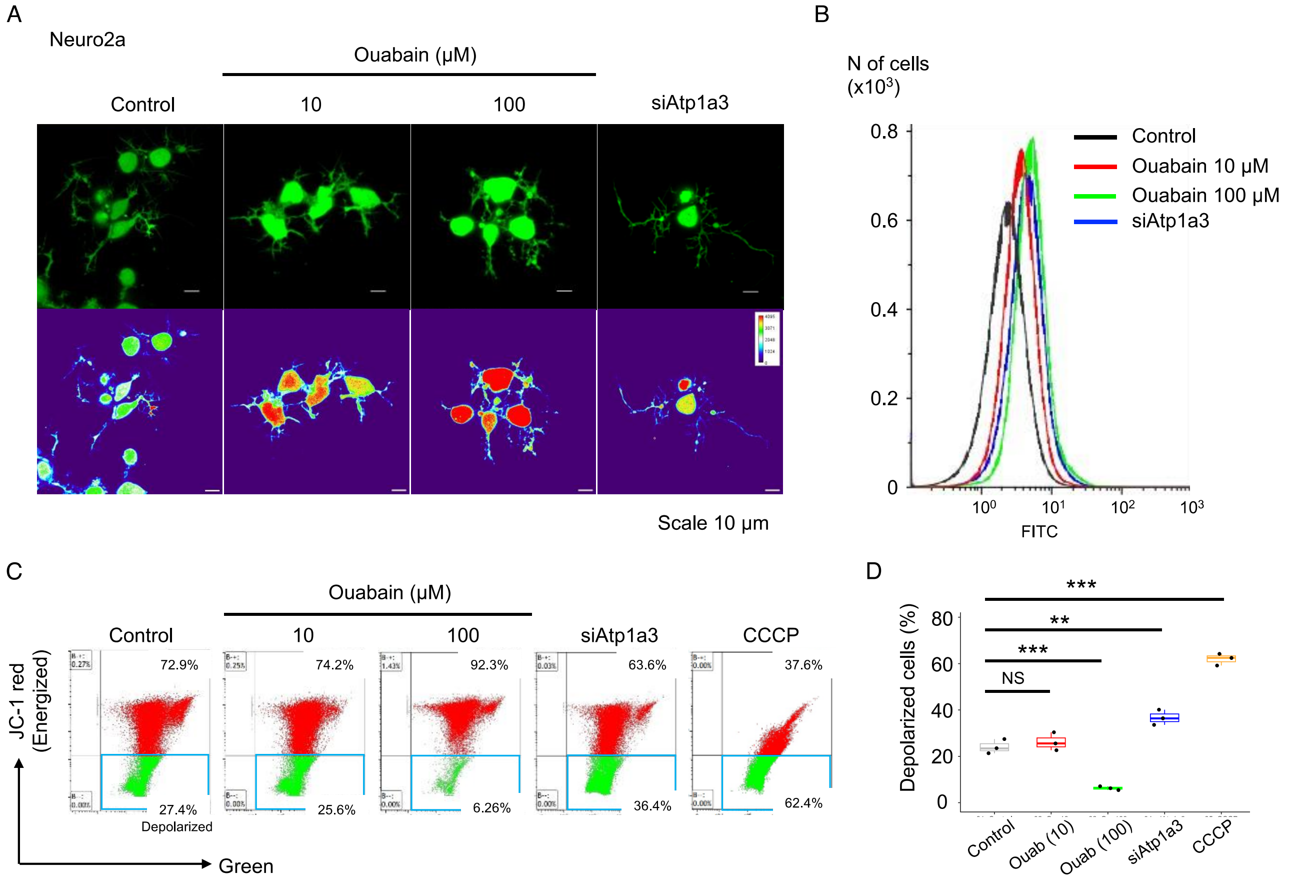

Fig. S6. Treatment of Neuro2a cells with the Na$^+$-K$^+$-ATPase inhibitor, ouabain.

A. Neuro2 cells were treated with 10 – 100 µM ouabain and siAtp1a3 for 48 hr prior to Fluo-4 and JC-1 assays. Note that both ouabain and siAtp1a3 treatments enhanced the calcium influx (high Fluo-4 signals) at the resting condition (**A, B**). Consistently, 100 µM ouabain-treated cells showed the lower rate (%) of cells with depolarized JC-1 signals than those of mock-treated cells (cells in light-blue squares in panel **C**). In contrast, siAtp1a3-treated cells showed the higher rate of cells with depolarized JC-1 signals. Panel **D** shows the quantitated results of panel **C**. CCCP was used as a reagent depolarizing the mitochondrial inner-membrane potential. **p<0.01, ***p <0.001 (Dunnett's test, n =3).

Disease Models & Mechanisms • Supplementary information

Neuro2a

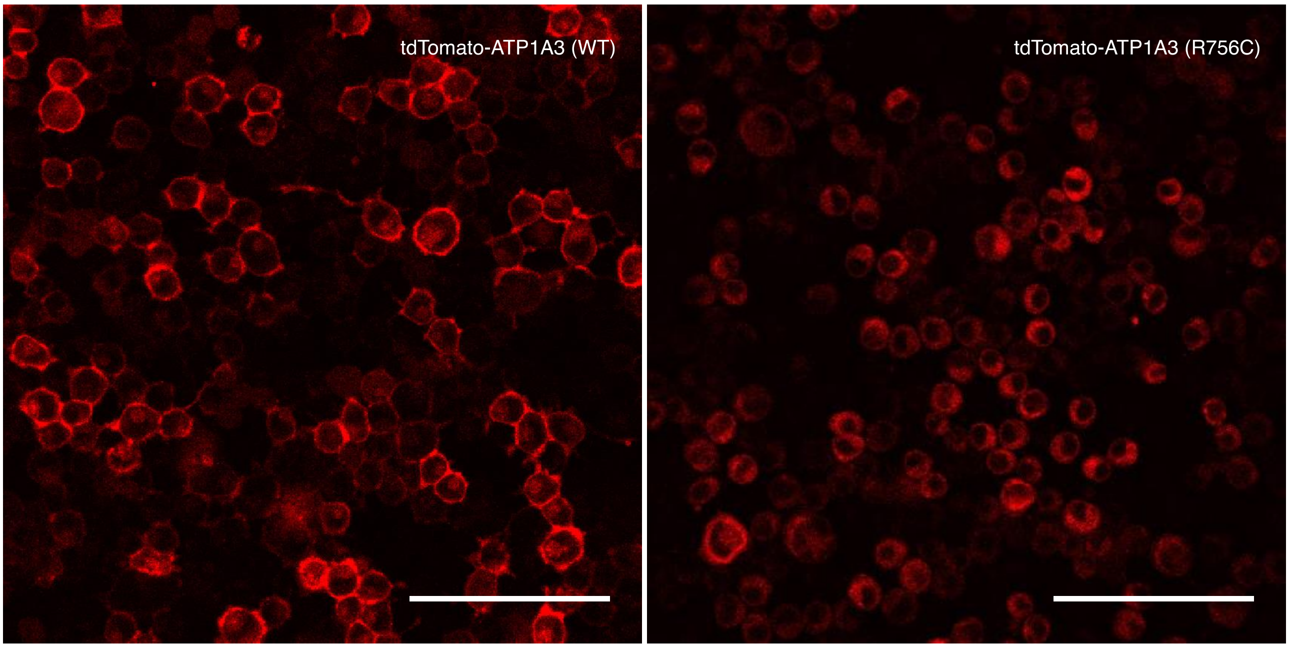

Scale, 100 μm

Fig. S7. Confocal microscopic images of Neuro2a cells expressing tdTomato-tagged ATP1A3. Stable cell lines were established using a cell sorter system. Panels show bright field and fluorescence images of Neuro2a cells expressing tdTomato-tagged wild-type (WT) and p.R756C full-length human ATP1A3.

Disease Models & Mechanisms • Supplementary information

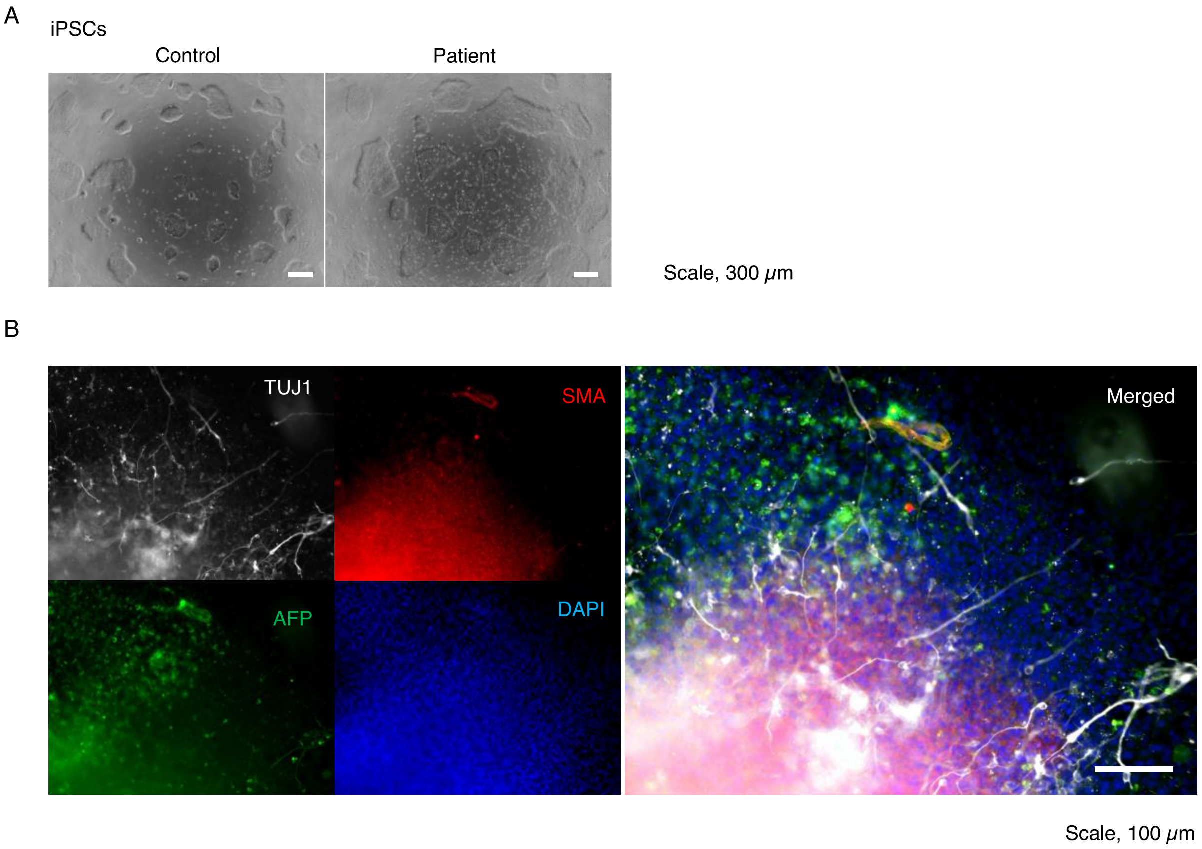

Fig. S8. Establishment of control and patient-derived iPSCs.

A. Phase contrast images of iPSCs from a healthy individual (Control) and a patient with p.R756C variant in *ATP1A3* (Patient).

B. Expression of three embryonic germ layers in iPSCs. TUJ1: beta-III tubulin (TUJ1) for ectoderm, SMA: smooth muscle actin for mesoderm, and AFP: alpha-fetoprotein for endoderm.

Disease Models & Mechanisms • Supplementary information

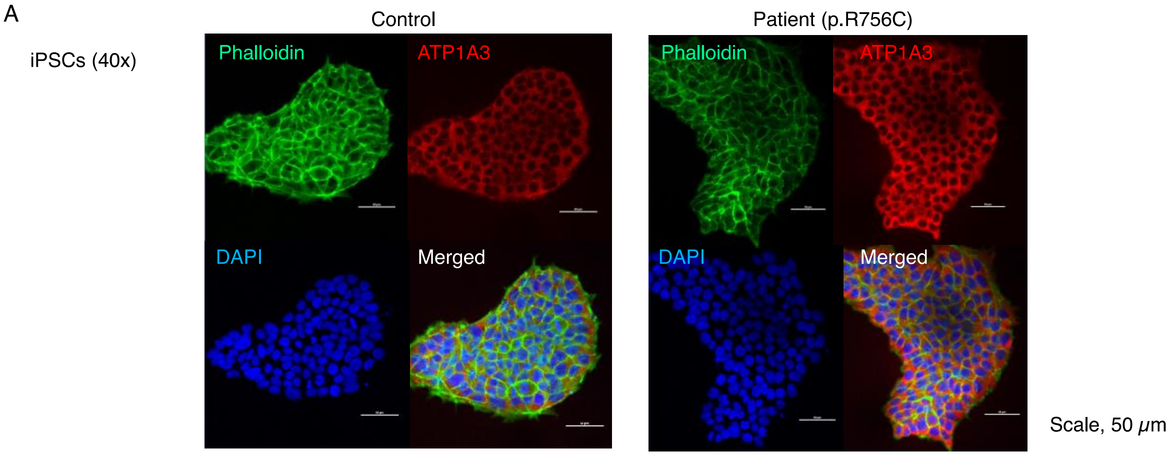

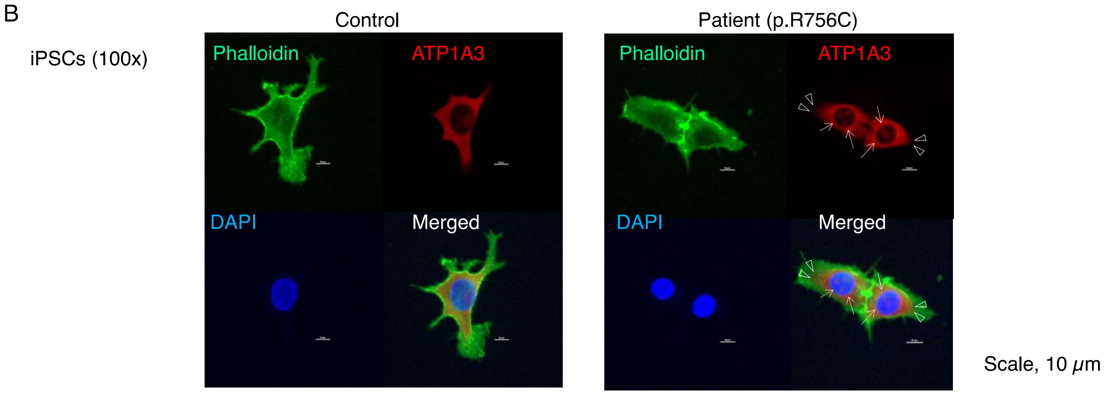

Fig. S9. Expression and distribution of ATP1A3 in iPSCs.

A. Confocal microscopic images show that the cytoplasmic expression of ATP1A3 in both control and patient iPSCs.

B. Higher ATPA1A3 (p.R756C) signals were observed in the perinuclear region (arrows) than the peripheral cytoplasmic regions (arrowheads) in the patient iPSCs. 40 and 100x, objective lends. Images were captured with 40× (A) and 100× (B) objective lens. DAPI (blue), phalloidin (green), and ATP1A3 (red) signals are shown as 3 channels and their merged views.

Disease Models & Mechanisms • Supplementary information

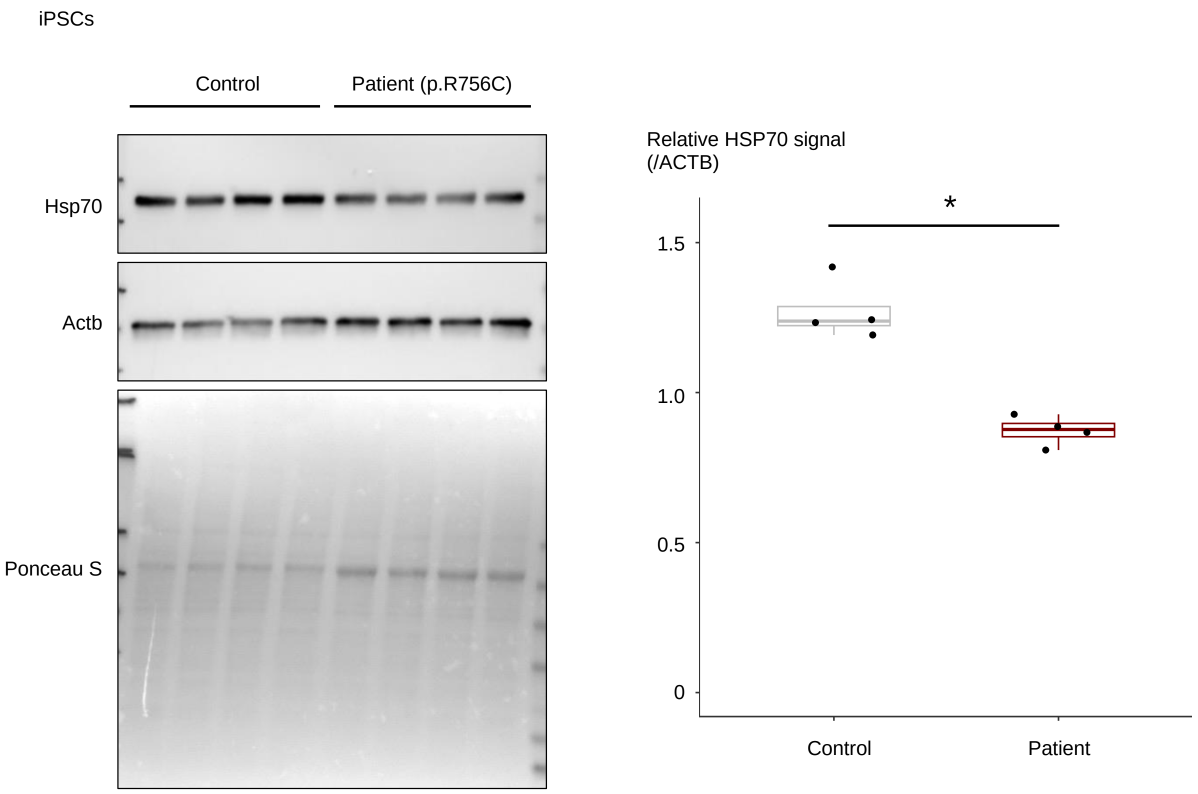

Fig. S10. Lower expression of HSP70 in the patient iPSCs than that in control iPSCs. Western blotting (left) shows lower expression of HSP70 in iPSCs from the patient with p.R756C compared to that in control iPSCs. Ponceau-S was used to visualize the amount of total protein loaded in each lane. Box-dot plots (right) show the quantitative measurement of HSP70 signals (relative ratio of HSP70/ACTB). *$p < 0.05$ (Wilcoxon's rank-sum test, $n = 4$ each).

Disease Models & Mechanisms • Supplementary information

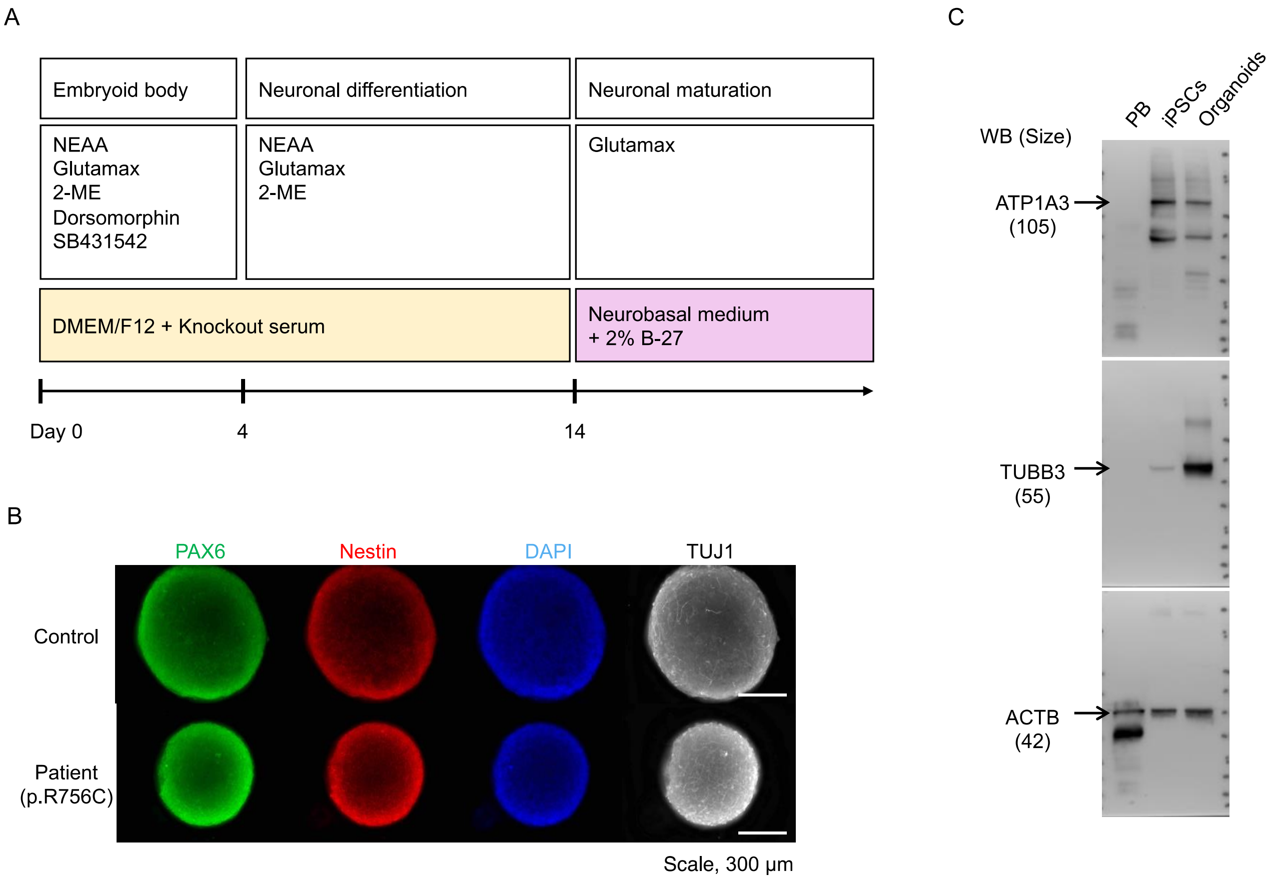

Fig. S11. Experimental protocol for neuronal differentiation.

A. Experimental procedure for neuronal differentiation and maturation from iPSCs using the SFEBq method.

B. Expression of PAX6, NESTIN, TUJ1 in neuro-spheroids on day 14 of differentiation *in vitro*. Similar levels of expression in these neuronal lineage markers were observed in neuro-spheroids from a healthy individual (Control) and a patient with p.R756C variant in *ATP1A3* (Patient). Monolayer neurons were prepared on 56 days of differentiation *in vitro* by placing these neuro-spheroids onto laminin 511-E8-coated coverslips.

C. Western blotting of peripheral blood (PB) mononuclear cells, iPSCs and neuronally differentiated spheroids at day 60 from a healthy adult (Control in panel B). Specific bands of ATP1A3, TUBB3 (Tuj1/Tubulin β3) and ACTB (actin-β) are shown in the corresponding panel (arrows).

Disease Models & Mechanisms • Supplementary information

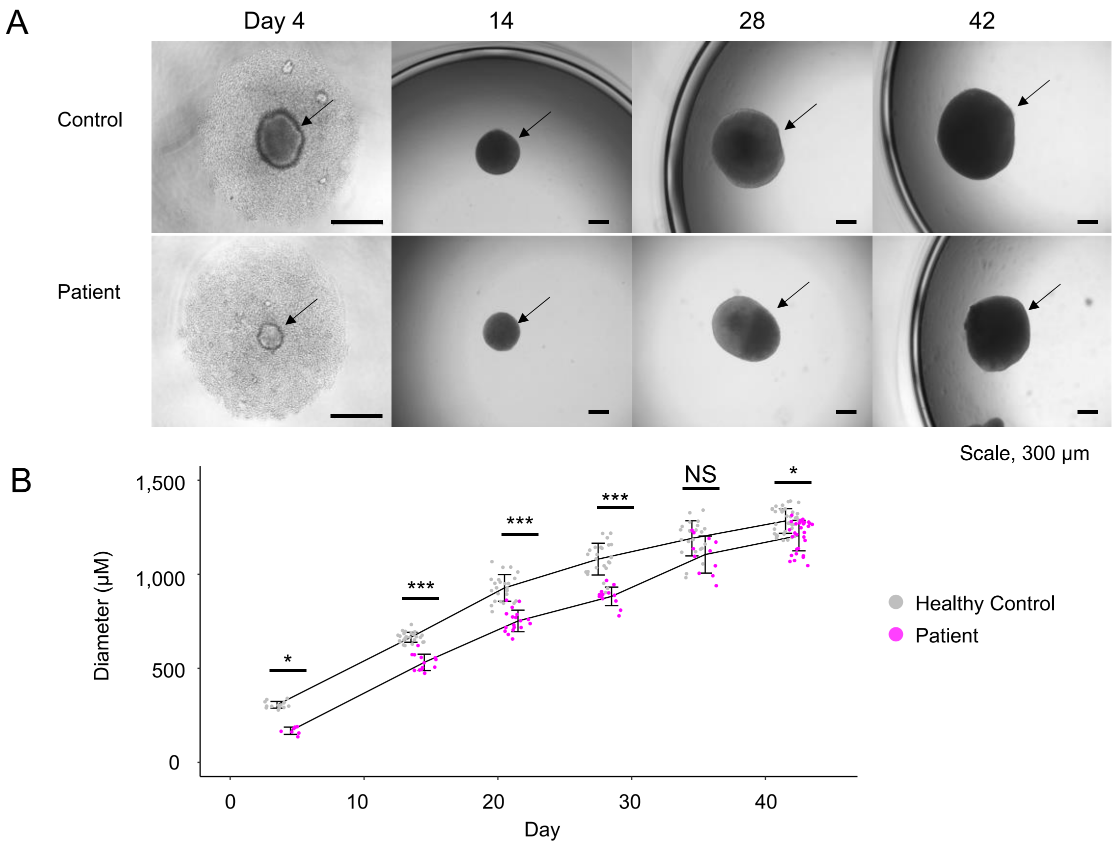

Fig. S12. Differential growth of neuro-spheroids from control and patient's iPSCs.

A. Bright field images of iPSCs during the time course of spheroid assembly. Panels show the increasing size of neuro-spheroids (arrows) were observed according to the progress in differentiation. Control, iPSCs from a healthy individual; Patient, iPSCs from a patient with p.R756C variant in *ATP1A3*.

B. Quantitative data of panel A. Mean ±SD values are shown in lines and error bars. Color dots indicate individual measurements. *p <0.05, ***p <0.001, NS, not significant (n = 14–37 for control [gray] and n = 7–30 for patient [pink]; Tukey's HSD).

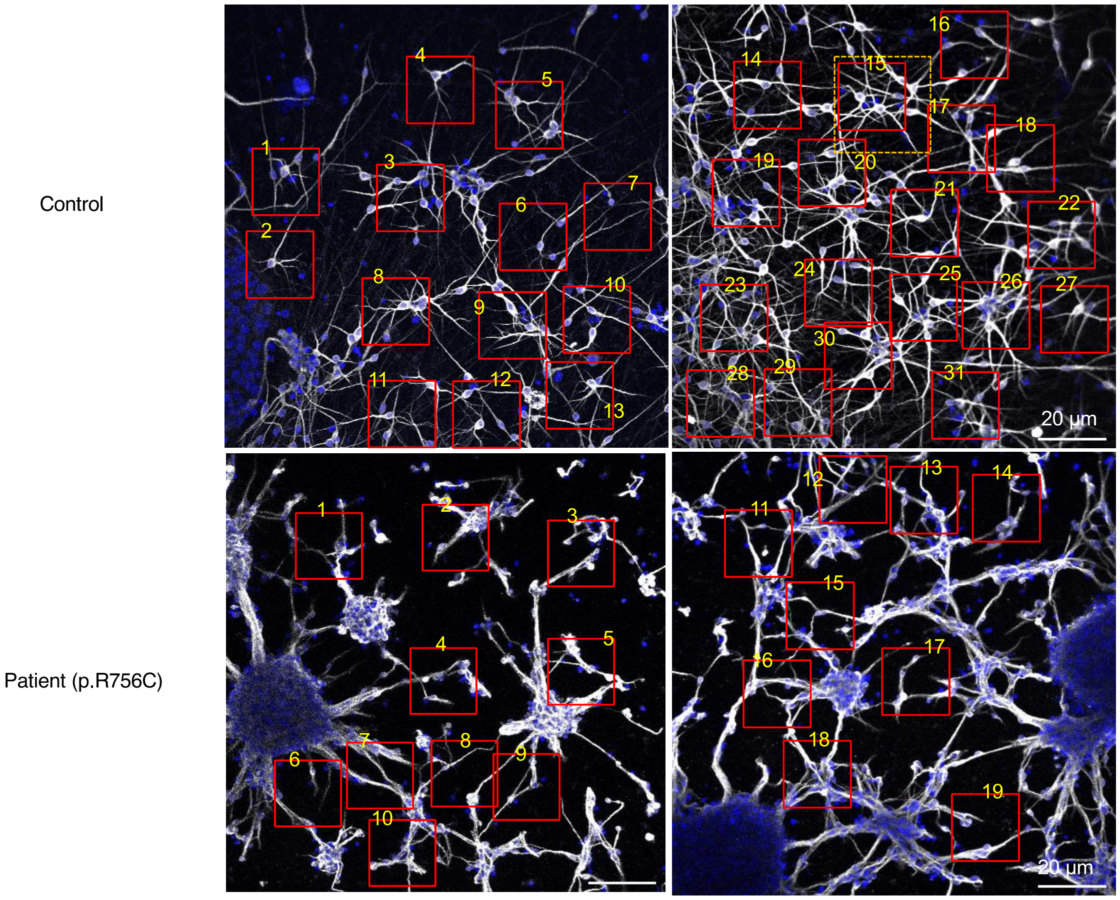

Fig. S13. Original images for counting the neurite outgrowth per cell.

Whole microscopic images show the morphological difference between neurons differentiated from control iPSCs (WT) and the patient iPSCs (p.R756C). Annotated squares indicate the sequential region of interest (ROI, control: #1 – 33 and patient: #1 – 19). The number of neurites per cell was counted in each ROI. Only MAP2 (white) and DAPI (blue) signals are shown for simplicity. The orange-dashed line in the right-upper panel indicates the region selected for the high-resolution image of control neurons (**Fig. 7A**). The high-resolution image of the patient neurons (p.R756C, Fig. 7A) was captured from an independent region from those in the lower two panels.

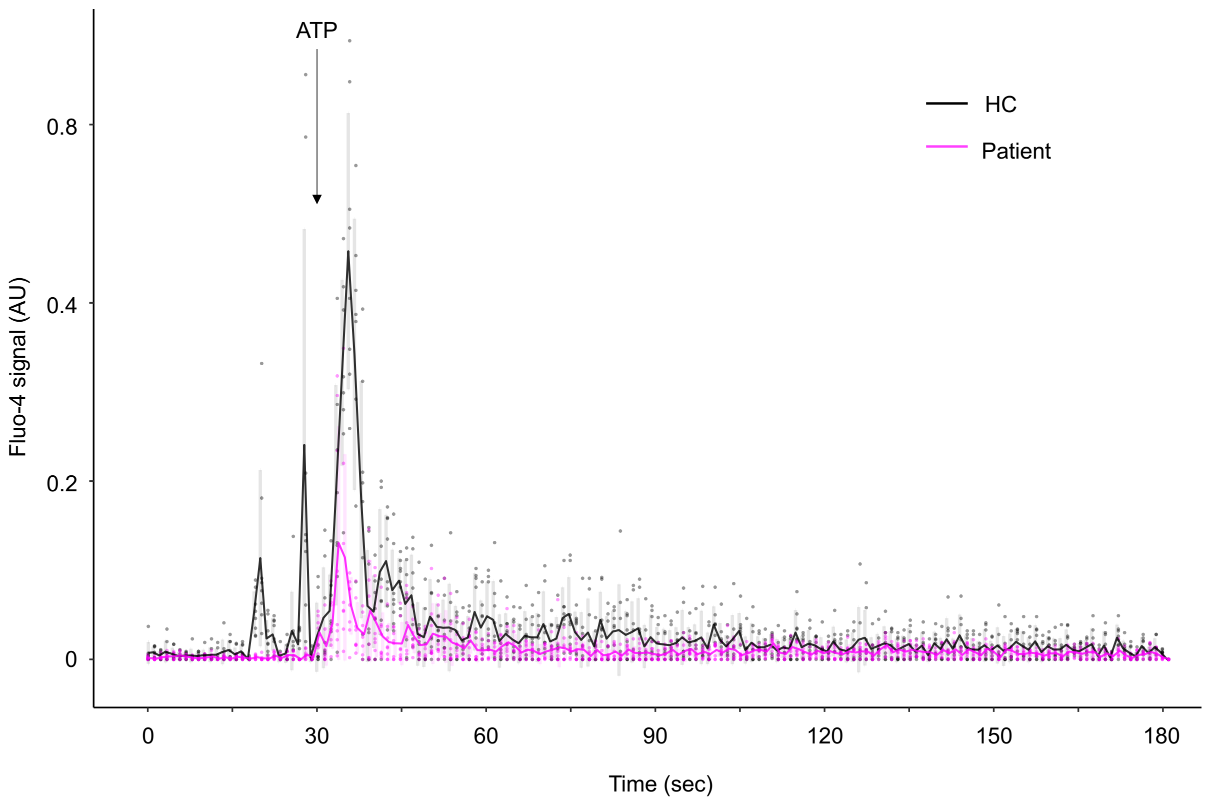

Fig. S14. Fluo-4 signal before and after stimulation with ATP.

Relative fluorescence signals of Fluo-4 were recorded for 3 min. ATP (100 µM) was added at time 30 sec. Mean ± SD (n = 8) values are shown as lines and error bars. Monolayer neurons from control and patient iPSCs were used for this analysis.

Disease Models & Mechanisms • Supplementary information

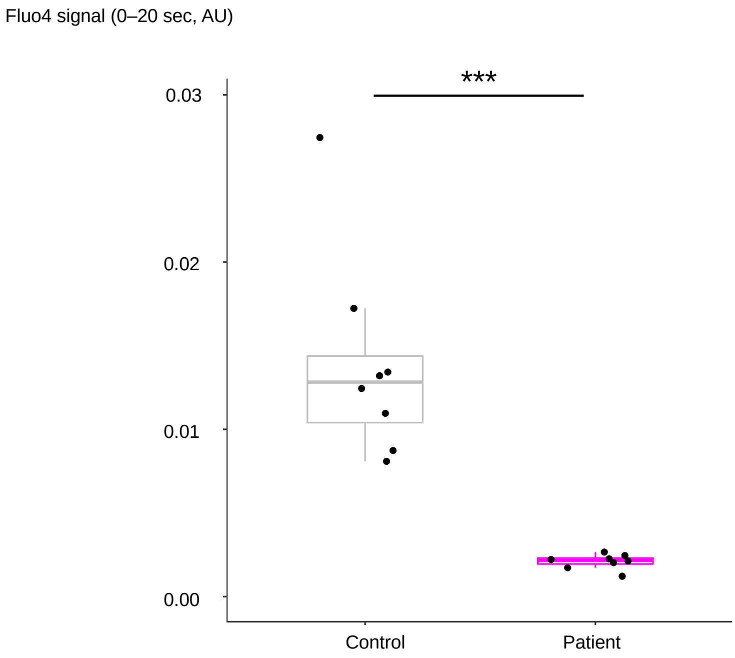

Fig. S15. Lower Fluo-4 signals in p.R756C neurons than those in controls during the resting condition. Fluo-4 signals were traced in control and patient iPSC-derived neurons before stimulation with ATP (0–20 s; **Fig. S14**). Quantitative measurements of the Fluo-4 signals in each neuron are presented asbox-dot plots. *p <0.05 (Wilcoxon's rank-sum test, n = 8 each).

Disease Models & Mechanisms • Supplementary information

Table S1. Summary of 136 ICL-binding proteins

Band	Accession	Gene	Description	Score[a]	Coverage	# Unique Peptides	#AA[b]	MW [kDa]
1	Q7TSC1	Prrc2a	Protein PRRC2A	89.21	26.78	22	2158	229.1
2	A2AVJ7	Rrbp1	Ribosome binding protein 1	120.90	24.39	6	1464	158.3
2	Q05D44	Eif5b	Eukaryotic translation initiation factor 5B	87.61	38.24	23	1216	137.5
2	E9QP99	Golga3	Golgin subfamily A member 3	84.95	30.73	27	1487	167.2
2	Q8VDD5	Myh9	Myosin-9	71.18	20.92	22	1960	226.2
2	B9EIU1	Eprs	Glutamyl-prolyl-tRNA synthetase	65.64	22.35	22	1512	169.9
2	Q64511	Top2b	DNA topoisomerase 2-beta	52.80	8.13	2	1612	181.8
3	E9QNN1	Dhx9	ATP-dependent RNA helicase A	351.58	60.84	72	1384	149.6
3	Q6P9L6	Kif15	Kinesin-like protein KIF15	203.64	56.67	67	1387	160.0
3	Q61595	Ktn1	Kinectin	135.03	42.50	2	1327	152.5
3	Q7TPV4	Mybbp1a	Myb-binding protein 1A	117.33	40.92	47	1344	151.9
3	Q6ZPS0	Rrbp1	MKIAA1398 protein (Fragment)	89.60	46.52	1	804	90.3
3	Q01320	Toop2a	DNA topoisomerase 2-alpha	87.10	34.42	37	1528	172.7
3	Q6PDH0	Phldb1	Pleckstrin homology-like domain family B member 1	65.42	26.11	26	1371	150.0
3	Q9EPU0	Upf1	Regulator of nonsense transcripts 1	61.84	33.45	27	1124	123.9
3	O70305	Atxn2	Ataxin-2	60.72	21.79	19	1285	136.4
4	D3Z2J3	Dhx30	Putative ATP-dependent RNA helicase DHX30	261.01	61.55	64	1186	132.9
4	Q7TQH0	Atxn2l	Ataxin-2-like protein	227.44	43.57	2	1049	110.6
4	J3QNB1	Larp1	La-related protein 1	214.98	66.14	64	1072	121.0
4	Q6A0A9	Fam120a	Constitutive coactivator of PPAR-gamma-like protein 1	120.57	40.65	35	1112	121.6
4	Q6PB66	Lrpprc	Leucine-rich PPR motif-containing protein, mitochondrial	89.76	33.19	38	1392	156.5
4	B7ZW94	Fam120c	Family with sequence similarity 120, member C	70.92	27.59	23	1091	119.6
4	G5E866	Sf3b1	Splicing factor 3B subunit 1	69.76	33.28	29	1304	145.7
4	Q8BKF1	polrmt	DNA-directed RNA polymerase, mitochondrial	69.08	28.17	23	1207	136.6
4	Q9Z1Q9	Vars	Valine--tRNA ligase	64.09	27.00	23	1263	140.1
4	Q8CG48	Smc2	Structural maintenance of chromosomes protein 2	50.27	28.21	27	1191	134.2
5	P52479	Usp10	Ubiquitin carboxyl-terminal hydrolase 10	183.37	29.17	16	792	87.0
5	Q60865	Caprin1	Caprin-1 OS=Mus musculus	158.08	26.03	16	707	78.1
5	G3XA10	Hnrnpu	Heterogeneous nuclear ribonucleoprotein U	130.86	38.59	23	793	86.8
5	P08730	Krt13	Keratin, type I cytoskeletal 13	111.85	23.11	6	437	47.7
5	P50446	Krt6a	Keratin, type II cytoskeletal 6A	82.81	22.24	4	553	59.3
5	Q3U868	Parp1	Putative uncharacterized protein (Fragment)	63.38	25.80	17	973	107.8
5	A2A513	Krt10	Keratin 10	61.86	12.66	5	561	57.0

Disease Models & Mechanisms • Supplementary information

5	Q3TTX0	Matr3	Putative uncharacterized protein	51.89	31.21	18	846	94.6
5	Q922U2	Krt5	Keratin, type II cytoskeletal 5	51.22	11.21	1	580	61.7
6	Q8VIJ6	Sfpq	Splicing factor, proline- and glutamine-rich	1325.52	64.09	51	699	75.4
6	Q3TA75	Fxr2	Putative uncharacterized protein (Fragment)	185.57	68.09	4	655	70.5
7	Q8R0E8	Atp1a3	Atp1a3 protein (Fragment)	1454.80	52.75	42	745	82.4
7	Q91VR5	Ddx1	ATP-dependent RNA helicase DDX1	364.57	70.41	54	740	82.4
7	Q3UYV9	Ncbp1	Nuclear cap-binding protein subunit 1	130.78	46.33	30	790	91.9
7	Q5F2E7	Nufp2	Nuclear fragile X mental retardation-interacting protein 2	117.08	49.28	27	692	75.6
7	Q6P5B5	Fxr2	Fragile X mental retardation syndrome-related protein 2	107.46	56.08	3	674	74.2
7	P29341	Pabpc1	Polyadenylate-binding protein 1	101.26	50.47	21	636	70.6
7	Q8CJG0	Eif2c2	Protein argonaute-2	72.38	44.42	25	860	97.2
7	B7ZWF1	Ddx3x	Ddx3x protein	63.42	45.08	22	661	73.0
7	Q8CAQ8	Immt	Mitochondrial inner membrane protein	62.31	49.67	21	757	83.8
7	Q8BML9	Qars	Glutaminyl-tRNA synthetase	57.59	36.52	22	775	87.6
7	Q91W50	Csde1	Cold shock domain-containing protein E1	56.90	39.85	21	798	88.7
7	Q91W18	Tdrd3	Tudor domain-containing protein 3	55.11	35.13	19	743	82.2
7	Q03141	Mark3	MAP/microtubule affinity-regulating kinase 3	53.19	34.40	15	753	84.3
8	P63017	Hspa8	Heat shock cognate 71 kDa protein	640.91	74.30	31	646	70.8
8	Q9D4E6	Pabpc6	Protein Pabpc6	453.81	22.55	1	643	71.0
8	P97855	G3bp1	Ras GTPase-activating protein-binding protein 1	368.41	63.01	21	465	51.8
8	Q792Y0	Pabpc2	Poly(A)-binding protein testis-specific isoform	275.45	27.86	1	603	66.4
8	B7U582	Hspa2	Heat shock protein 70-2	238.64	21.01	1	633	69.7
8	Q542W3	G3bp2	GTPase activating protein (SH3 domain) binding protein 2	179.35	59.24	1	449	50.8
8	Q3THB3	Hnrnpm	Heterogeneous nuclear ribonucleoprotein M	157.07	55.36	38	690	73.7
8	P20029	Hspa5	78 kDa glucose-regulated protein	126.21	47.63	22	655	72.4
8	P38647	Hspa9	Stress-70 protein, mitochondrial	100.16	50.96	30	679	73.4
8	E9QAT0	Fmr1	Fragile X mental retardation protein 1 homolog	67.56	49.92	16	589	66.1
8	P03975	Iap	IgE-binding protein	67.18	37.34	1	557	62.7
8	Q8C2Q3	Rbm14	RNA-binding protein 14	66.55	36.47	21	669	69.4
8	Q80WJ7	Mtdh	Protein LYRIC	59.15	46.80	19	579	63.8
9	Q61656	Ddx5	Probable ATP-dependent RNA helicase DDX5	553.14	65.15	37	614	69.2
9	Q571F9	G3bp1	MKIAA4115 protein (Fragment)	351.96	61.19	23	505	56.1
9	E9PUK3	Fnbp1l	Formin-binding protein 1-like	218.35	69.51	40	551	63.9
9	Q501J6	Ddx17	Probable ATP-dependent RNA helicase DDX17	191.38	34.92	10	650	72.4
9	P61979	Hnrpk	Heterogeneous nuclear ribonucleoprotein K	188.63	63.28	26	463	50.9
9	Q61584	Fxr1	Fragile X mental retardation syndrome-related protein 1	186.06	59.08	28	677	76.2
9	Q925I1	Atad3	ATPase family AAA domain-containing protein 3	121.45	62.44	36	591	66.7

Disease Models & Mechanisms • Supplementary information

9	Q3UDS0	Hspa8	Putative uncharacterized protein (Fragment)	119.45	64.20	29	553	60.8
9	A3KFU5	Pabpc4	Poly A binding protein, cytoplasmic 4 OS=Mus musculus GN=Pabpc4 PE=4 SV=1 - [A3KFU5_MOUSE]	67.43	31.54	6	631	69.4
9	Q9CPN8	Igf2bp3	Insulin-like growth factor 2 mRNA-binding protein 3	64.07	42.83	20	579	63.5
9	Q3UMT7	Hnrnpl	Putative uncharacterized protein	50.72	56.76	16	592	64.0
9	Q7TMK9	Syncrip	Heterogeneous nuclear ribonucleoprotein Q	50.65	37.56	15	623	69.6
10	Q3TFC2	Nono	Putative uncharacterized protein	1833.17	72.94	1	473	54.4
10	P20152	Vim	Vimentin	534.04	82.19	4	466	53.7
10	Q3TWV0	Vim	Putative uncharacterized protein	495.46	74.89	1	466	53.6
11	P70333	Hnrnph2	Heterogeneous nuclear ribonucleoprotein H2	349.10	51.00	5	449	49.2
11	P15331	Prph	Peripherin	327.55	81.26	39	475	54.2
11	P99024	Tubb5	Tubulin beta-5 chain	183.82	71.62	4	444	49.6
11	P68369	Tuba1a	Tubulin alpha-1A chain	177.30	64.30	2	451	50.1
11	P05213	Tuba1b	Tubulin alpha-1B chain	168.10	61.42	1	451	50.1
11	P68372	Tubb4b	Tubulin beta-4B chain	159.90	74.16	3	445	49.8
11	Q7TMM9	Tubb2a	Tubulin beta-2A chain	143.67	66.97	3	445	49.9
11	Q71V06	Ybx1	Y box transcription factor (Fragment)	134.02	71.91	16	299	33.5
11	Q8BK67	Rcc2	Protein RCC2	123.10	66.54	27	520	55.9
11	Q9ERD7	Tubb3	Tubulin beta-3 chain	115.81	52.00	5	450	50.4
11	Q8BGJ5	Ptbp1	MCG13402, isoform CRA_a	108.67	55.39	13	529	56.9
11	Q8BG81	Poldip3	Polymerase delta-interacting protein 3	68.33	53.81	20	420	46.1
11	Q8R326	Pspc1	Paraspeckle component 1	63.01	40.34	14	523	58.7
11	Q3TF87	Dars	Putative uncharacterized protein	58.72	53.09	22	501	57.1
11	Q68G78	Csda	Csda protein	57.48	49.83	1	299	31.8
11	Q9CY58	Serbp1	Plasminogen activator inhibitor 1 RNA-binding protein	54.52	47.67	1	407	44.7
11	Q9JKB3	Csda	DNA-binding protein A	54.51	39.61	1	361	38.8
11	Q3UMP4	Serbp1	Putative uncharacterized protein	53.91	48.13	1	401	44.0
12	Q9D8E6	Rpl4	60S ribosomal protein L4	263.87	66.59	35	419	47.1
12	Q921V6	Trim21	Trim21 protein	125.01	50.21	27	470	54.1
12	Q9Z2X1	Hnrnpf	Heterogeneous nuclear ribonucleoprotein F	107.44	53.98	14	415	45.7
12	Q91VC3	Eif4a3	Eukaryotic initiation factor 4A-III	92.84	63.75	19	411	46.8
12	Q91VM5	Rbmxl1	RNA binding motif protein, X-linked-like-1	71.04	62.11	29	388	42.1
12	P10126	Eef1a1	Elongation factor 1-alpha 1	63.67	48.48	21	462	50.1
12	Q8C2Q7	Hnrnph1	Heterogeneous nuclear ribonucleoprotein H	62.70	42.37	4	472	51.2
12	P32067	Ssb	Lupus La protein homolog	52.92	55.90	18	415	47.7
13	P60710	Actb	Actin, cytoplasmic 1	193.90	72.00	0	375	41.7
13	Q3UGS0	Actb	Putative uncharacterized protein	190.41	72.00	1	375	41.7

Disease Models & Mechanisms • Supplementary information

13	Q8BVA9	Elavl4	ELAV (Embryonic lethal, abnormal vision, Drosophila)-like 4 (Hu antigen D)	105.62	63.68	8	380	41.7
13	Q3UR02	Elavl2	ELAV (Embryonic lethal, abnormal vision, Drosophila)-like 2 (Hu antigen B)	94.98	58.77	7	359	39.5
13	Q8BFZ3	Actbl2	Beta-actin-like protein 2	78.97	30.05	1	376	42.0
13	O88986	Gcat	2-amino-3-ketobutyrate coenzyme A ligase, mitochondrial	78.46	58.89	18	416	44.9
13	A2AL12	Hnrnpa3	Heterogeneous nuclear ribonucleoprotein A3	66.88	54.09	19	318	34.5
14	P49312	Hnrnpa1	Heterogeneous nuclear ribonucleoprotein A1	139.85	58.75	19	320	34.2
14	O88569	Hnrnpa2b1	Heterogeneous nuclear ribonucleoproteins A2/B1	126.64	62.32	19	353	37.4
14	Q5FWB6	Rplp0	MCG17387, isoform CRA_a	68.00	64.35	17	317	34.2
14	P47911	Rpl6	60S ribosomal protein L6	65.29	54.39	23	296	33.5
14	E9Q7H5	Gm8991	Uncharacterized protein	64.34	54.33	18	300	32.6
14	P70372	Elavl1	ELAV-like protein 1	50.63	45.40	13	326	36.1
15	Q4V9X9	Rpl23a	Rpl23a protein (Fragment)	94.49	56.13	13	155	17.6
15	P62082	Rps7	40S ribosomal protein S7	94.21	72.68	16	194	22.1
15	Q6ZWV3	Rpl10	60S ribosomal protein L10	90.00	78.50	19	214	24.6
15	Q6ZWN5	Rps9	40S ribosomal protein S9	88.52	76.80	18	194	22.6
15	Q80V08	Rpl17	Rpl17 protein (Fragment)	69.76	60.82	12	194	22.4
15	Q8BP67	Rpl24	60S ribosomal protein L24	51.45	40.76	8	157	17.8
16	P62281	Rps11	40S ribosomal protein S11	85.26	75.32	12	158	18.4
16	P61255	Rpl26	60S ribosomal protein L26	55.75	71.72	5	145	17.2
17	P62830	Rpl23	60S ribosomal protein L23	145.43	72.14	15	140	14.9
17	P62245	Rps15a	40S ribosomal protein S15a	117.04	77.69	15	130	14.8
17	P14131	Rps16	40S ribosomal protein S16	97.56	78.08	15	146	16.4
17	O70569	Rps14	Ribosomal protein S14	58.97	43.05	10	151	16.3
18	P62892	Rpl39	60S ribosomal protein L39	51.15	37.25	2	51	6.4
19	P23246	Sfpq	Splicing factor, proline- and glutamine-rich	1826.49	58.27	51	707	76.1
19	P04264	Krt1	Keratin, type II cytoskeletal 1	94.01	41.15	18	644	66.0
19	P35527	Ktr9	Keratin, type I cytoskeletal 9	67.45	60.51	14	623	62.0
19	P19338	Ncl	Nucleolin	61.78	21.55	13	710	76.6
19	Q15233	Nono	Non-POU domain-containing octamer-binding protein	59.60	24.42	5	471	54.2
19	P35908	Krt2	Keratin, type II cytoskeletal 2 epidermal	58.33	46.79	15	639	65.4

a. Proteins only with high-confident score (>50) are presented.
b. AA, amino acid

Disease Models & Mechanisms • Supplementary information

Table S2. Antibodies and oligonucleotides used in this study.

Reagents	Vendor	Catalogue #, dilution
Antibodies		
Anti-4EBP1	Cell Signaling Technol	9452, 1:1000
Anti-Actb (HRP-conjugated)	Abcam	ab6276, 1:10000
Anti-Atp1a3	Santa Cruz Biotechnol	sc-374050, 1:200
Anti-CYFIP1	Millipore	AB6046, 1:1000
Anti-EIF4G	Cell Signaling Technol	2498, 1:1000
Anti-FMRP	Cell Signaling Technol	7104, 1:1000
Anti-GFP mAb-Magnetic beads	MBL	D153-11, 1:1000
Anti-GFP (mouse)	Clontech	632380, 1:1000
Anti-GFP (chicken)	Abcam	ab13970, 1:1000
Anti-Hsp70	Abcam	ab2787, 1:1000
Anti-MAP2	Abcam	ab5392, 1:1000
Anti-Nestin	BD Biosciences	556309, 1:1000
Anti-PABPC1	Cell Signaling Technol	4992, 1:1000
Anti-PAX6	Abcam	ab5790, 1:1000
Anti-PKR	Cell Signaling Technol	12297, 1:1000
Anti-PSD95	Cell Signaling Technol	3450, 1:1000
Anti-RPS6	Cell Signaling Technol	2217, 1:1000
Anti-phospho-RPS6	Cell Signaling Technol	5364, 1:1000
Anti-DsRed (that also detects tdTomato)	Clontech	632394, 1:1000
Anti-TOM20	Cell Signaling Technol	42406, 1:1000
Anti-TSC1	Cell Signaling Technol	4906, 1:1000
Anti-TSC2	Cell Signaling Technol	4308, 1:1000
Anti-TUBB3	Abcam	ab18207, 1:1000
Peroxidase AffiniPure goat anti-mouse antibody	Jackson Immunoresearch	115-035-174, 1:5000
Peroxidase IgG fraction monoclonal mouse anti-Rabbit IgG, light chain specific	Jackson Immunoresearch	211-032-171, 1:5000
Recombinant DNA		
pEGFP-N2	Clontech	6081-1
ptdTomato-N1	Takara Bio Inc.	632532
pEGFP-parkin WT	Addgene	45875
PCR primers and siRNAs		
Mouse *Actb*: CTAAGGCCAACCGTGAAAG; ACCAGAGGCATACAGGGACA		
Mouse *Atp1a3*: AGGGTTTGACCCACAGCAAA; AGGCCAGGAAGCAGAGGATA		
Mouse *Ap2b1*: GAGGAAGGAGGCTGTGAAGA; TCTGGCTGACTCTTGGCATA		
Mouse *Apc*: TGAGTGCCTTATGGAACCTGT; CTCCGGTAAGTGAGGGTGC		
Mouse *Arc*: AAGCTGGAGAACAACTTGGACGG; CCCCCAAGACTGATATTGCTGAG		
Mouse *Actb*: CTAAGGCCAACCGTGAAAG; ACCAGAGGCATACAGGGACA		
Mouse *Camk2a*: GGAGGGACACCACTACCTGA; GAGGCCAGCAACAGATTCTC		
Mouse *Ctnnb1*: GCAGCAGCAGTTTGTGGA; TGTGGAGAGCTCCAGTACACC		
Mouse *Fmr1*: ACCAGTTGCGTTTGGAGAGA; AGCATTTGATGCTTCAGAATTAGT		
Mouse *Fus*: AGGCCTGGGTGAGAATGTTA; GCTGTCCCGTTTTCTTGTTT		
Mouse *Hnrnpa2b1*: ATGGCTGCAAGACCTCATTC; TAATTCCGCCAACAAACAGC		
Mouse *Map1b*: TGGGACACAAACCTGATTGA; AGGGTTGATCAGGACCACTG		
Mouse *Pkp4*: CGTGAAGGAGCAGGTGTTTA; GATTCTGCTCCAAGCCTACAT		
Mouse *Cdk5*: GCCCTACCCAATGTACCCAG; GAAGTAGGGGTGCTGCAAGG		

Disease Models & Mechanisms • Supplementary information

Mouse *Dkk1*: TCTCTATGAGGGCGGGAACA; TTTCGGCAAGCCAGACAGAT		
Mouse *Il10*: GGTTGCCAAGCCTTATCGGA; AATCGATGACAGCGCCTCAG		
Mouse *Hspbp1*: TGGCTATTACTGCGGGTTCT; CATCTGCTCCACCTCCTCTC		
Mouse *Hspa8*: GTCTGATCGGGCGTAGGTTT; TGGTAACGGTCTTTCCGAGG		
Mouse *Hsp90aa1*: CCTGACGGACCCCAGTAAAC; TATCTGCACCAGCCTGCAAA		
Mouse *Hsp90ab1*: CCAATGACTGGGAGGACCAC; TCAACCACACCGCGGATAAA		
Mouse *Hspa1a*: CAGCGAGGCTGACAAGAAGA; CTCCCTTGGGCGCCTG		
In-Fusion cloning of ICL *into* pEGFP-N2: GGACTCAGATCTCGAGGCCACCATGACGCTGACC; CCGCGGTACCGTCGACGGAGTCTCTCATTGACCAA		
In-Fusion cloning of *ATP1A3* into ptd-Tomato: CTCAAGCTTCGAATTCGCCACCATGGGGGACAAG; CCGCGGTACCGTCGACCCGTAGTAGGTTTCCTTCT		
siRNA to *Atp1a3* (si*Atp1a3*)	Thermo Fisher Scientific	#HSS100796
siRNA negative Control	Thermo Fisher Scientific	12935300

PCR primers (5' to 3') are shown as forward and reverse pairs from left to right.

Disease Models & Mechanisms • Supplementary information
